# PDGFR-alpha inhibits melanoma growth via CXCL10/IP-10: a multi-*omics* approach

**DOI:** 10.18632/oncotarget.12629

**Published:** 2016-10-13

**Authors:** Daniela D'Arcangelo, Francesco Facchiano, Giovanni Nassa, Andrea Stancato, Annalisa Antonini, Stefania Rossi, Cinzia Senatore, Martina Cordella, Claudio Tabolacci, Annamaria Salvati, Roberta Tarallo, Alessandro Weisz, Angelo M. Facchiano, Antonio Facchiano

**Affiliations:** ^1^ Istituto Dermopatico dell'Immacolata, IDI-IRCCS, Fondazione Luigi Maria Monti, Rome, Italy; ^2^ Dipartimento Ematologia, Oncologia e Medicina Molecolare, Istituto Superiore di Sanità, Rome, Italy; ^3^ Laboratory of Molecular Medicine and Genomics, Department of Medicine and Surgery,University of Salerno, Baronissi (SA), Italy; ^4^ Genomix4Life srl, Department of Medicine and Surgery, University of Salerno, Baronissi (SA), Italy; ^5^ National Research Council Institute of Food Science, Avellino, Italy

**Keywords:** cancer, omics, angiogenesis, miRNA, melanoma

## Abstract

Melanoma is the most aggressive skin-cancer, showing high mortality at advanced stages. Platelet Derived Growth Factor Receptor-alpha (PDGFR-alpha) potently inhibits melanoma- and endothelium-proliferation and its expression is significantly reduced in melanoma-biopsies, suggesting that melanoma progression eliminates cells expressing PDGFR-alpha. In the present study transient overexpression of PDGFR-alpha in endothelial (HUVEC) and melanoma (SKMel-28, A375, Preyer) human-cells shows strong anti-proliferative effects, with profound transcriptome and miRNome deregulation. PDGFR-alpha overexpression strongly affects expression of 82 genes in HUVEC (41 up-, 41 down-regulated), and 52 genes in SKMel-28 (43 up-, 9 down-regulated). CXCL10/IP-10 transcript showed up to 20 fold-increase, with similar changes detectable at the protein level. miRNA expression profiling in cells overexpressing PDGFR-alpha identified 14 miRNAs up- and 40 down-regulated, with miR-503 being the most down-regulated (6.4 fold-reduction). miR-503, miR-630 and miR-424 deregulation was confirmed by qRT-PCR. Interestingly, the most upregulated transcript (i.e., CXCL10/IP-10) was a validated miR-503 target and CXCL10/IP-10 neutralization significantly reverted the anti-proliferative action of PDGFR-alpha, and PDGFR-alpha inhibition by Dasatinb totally reverted the CXCL10/IP10 induction, further supporting a functional interplay of these factors. Finally, integration of transcriptomics and miRNomics data highlighted several pathways affected by PDGFR-alpha.

This study demonstrates for the first time that PDGFR-alpha strongly inhibits endothelial and melanoma cells proliferation in a CXCL10/IP-10 dependent way, *via* miR-503 down-regulation.

## INTRODUCTION

Cutaneous melanoma is the most aggressive skin cancer. Despite recent relevant therapeutic progresses, melanoma has still a poor prognosis at advanced clinical phases and contributes for the vast majority to skin cancers-related mortality. Multiple factors have been involved in the melanoma pathogenesis. A clear role has been identified for the BRAF mutational state along with sun exposure [1], intentional tanning [2] and exposure to other environmental pollutions such as insecticides and occupational exposures [3]. Increasing evidence is being collected indicating a role of the immunity, which appears to be impaired at least to some extent in melanoma [4]. To this regard, it should be highlighted that one of the recent advances in melanoma therapy refers to Ipilimumab [5], a CTLA-4 inhibitor acting as an immune response activator. Immune response is under the control of a complex molecular-network, including cytokines and chemokines [6], vitamins, interferon and interferon-induced proteins such as Interferon (IFN)-γ-induced protein 10 (CXCL10/IP-10) [7, 8]. Inflammatory response is a key factor controlling initiation and progression of melanoma as well as other tumors [9, 10]; it is known to contribute to tumor development and anti-inflammatory agents are known to inhibit cancer growth, representing a class of novel potential anti-melanoma drugs [11]. Inflammation and immune response cooperate at different molecular levels to maintain the homeostasis and to control cancer development [12]. Several factors controlling inflammation and immune response have been identified to play regulatory actions in melanoma, such as Platelet Activating Factor (PAF) [9], microphthalmia-associated transcription factor (MITF) [13], granulocyte-macrophage colony-stimulating factor [14], TNF-alpha [15], transforming growth factor [16]. CXCL10/IP-10 has a clear controlling role on immune response and inflammation [17–20] and has been recently found to be involved in liver and renal cancer development [21, 22]. It has been reported to inhibit melanoma growth and angiogenensis [23–26] and melanoma represses its expression in a nitric oxide-related manner [27].

Several growth factors, including Platelet Derved Growth Factors (PDGFs), have been shown to play a critical role in controlling angiogenesis as well as proliferation and metastatic potential of melanoma [28–32]. The PDGF family exerts mitogenic and chemotactic actions in many cell types and with different potency. Such factors are present in 5 homo- and hetero-dimeric complexes (PDGF-AA, PDGF-BB, PDGF-AB, PDGF-CC, PDGF-DD) and bind to 3 dimeric receptors, namely PDGFRalpha/alpha (binding PDGFAA, PDGF-AB, PDGF-CC), PDGFRbeta/beta (binding PDGF-BB and PDGF-DD) and PDGF-Ralpha/beta (binding PDGF-AB, PDGF-BB, PDGF-CC, PDGF-DD) [33, 34]. As recently highlighted, the specific role such receptors play in cancer development and progression has still to be largely clarified [35]. The stimulatory role of PDGF-BB and PDGFR-beta has been demonstrated in several cells and tissues, including melanoma [36–40]. Non-selective inhibitors of both PDGFR-beta and PDGFR-alpha, such as imatinib and dasatinib, are known to partially inhibit melanoma growth, but the role of the alpha or beta receptors has not been fully clarified yet [41–43].

PDGF-AA and PDGFR-alpha are less potent mitogens, with contrasting- even inhibitory-effects in different cells and tissues, including endothelial and melanoma cells, as demonstrated in previous studies by ours and other laboratories [31, 44–46]. A recent study highlighted an inhibitory effect of PDGFR-alpha in ulcer healing, indicating a PDGFR-alpha mediated anti-angiogenesis effect [47].

We have previously shown that PDGF-AA inhibits PDGF-BB angiogenic effects [28], and showed a direct interplay of Fibroblast Growth Factor-2 (FGF2) and PDGFs pathways, by demonstrating high-affinity binding of FGF-2 to PDGF-BB [48] [and FGF-receptor/PDGFR-alpha heterodimers formation [30]. We have also shown that PDGFR-alpha inhibits cell-growth both *in vitro* and *in vivo*, in endothelial- [29] as well as melanoma cells [31]. Such evidence shows a relevant anti-proliferation activity of PDGFR-alpha in melanoma; its expression has been found to be lost in human melanoma biopsies, likely due to a selection pressure acting to eliminate inhibitory factors. Further, over-expressing PDGFR-alpha in melanoma reduces proliferation, inhibits DNA synthesis and increases apoptosis, cell cycle arrest and pRb dephosphorylation [31].

In the present study we better elucidate the molecular mechanisms underlying the PDGFR-alpha mediated anti-melanoma effect. Further, transcriptomics and miRNomics data from human melanoma and endothelial cells over-expressing PDGFR-alpha were combined in a multi-*omics* analysis, leading to the identification of pathways and functions involved in the anti-melanoma action of PDGFR-alpha overexpression.

## RESULTS

### PDGFR-alpha overexpression inhibits endothelial and melanoma cells *in vitro* proliferation

**Figure 1:**
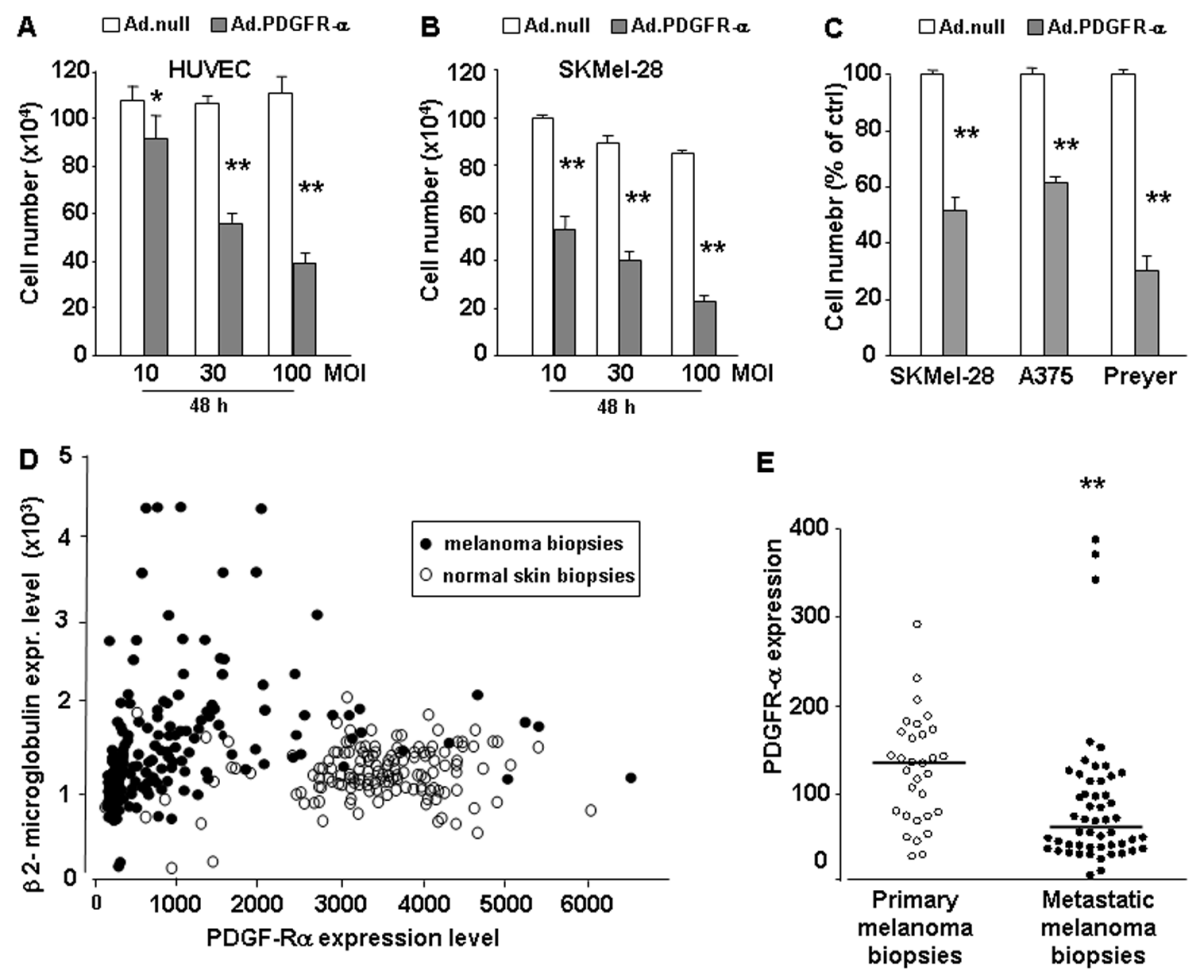
Effect of PDGFR-alpha overexpression on endothelial (HUVEC) and melanoma (SKMel-28) cells proliferation Proliferation of HUVEC and SKMel-28 infected with Ad-vector codingfor PDGFR-alpha. **A, B.**: Dose-dependent effects of 10, 30, and 100 MOI AdCMV.PDGFR-alpha infection; 10% FCS–induced proliferation at 48 hours. *p value < 0.05 and **p value <0.01 versus AdCMV.null (cell number at T = 0 corresponds to 7.5 ×10^5^). Data are reported as mean ± SD of 3 independent experiments. **C.** Effects of AdCMV.PDGFR-alpha infection in SKMel-28, A375 and Preyer cell lines. Data are reported as % of control **D.** Expression of PDGFR-alpha (X axis) vs expression of the housekeeping gene beta 2-microglobulin (Y axis) in 208 melanoma biopsies (black spots) and 147 normal skin biopsies. Expression data were derived from ist.medisapiens.com site. Supplementary Figure 2SA-2SB reports correlation plots of PDGFR-alpha with other housekeeping genes, namely Tubulin Beta 1 gene (Supplementary Figure 2SA) and Actin beta gene (Figure 2SB). In all cases PDGFR-alpha is strongly reduced in melanoma samples vs normal skin samples. **E.** Mean PDGFR-alpha expression in 31 primary human melanoma biopsies and in 52 metastatic human melanoma biopsies is significantly reduced (p = 0.0002). Analysis carried onto GEO database, GDS3966 dataset (Xu 2008) (http://www.ncbi.nlm.nih.gov/pubmed). Expression values are reported. Graphing the “ranks values” of PDGFR-alpha (instead of the “uncorrected values”) does not modify the significant reduction in metastatic vs primary samples (0.58 vs 0.73, p = 0.0004). Beta 2-microglobulin, beta actin or tubulin beta1 ranks are very stable, invariantly 99 or 100 in all melanoma samples, independently from the “primary” or “metastatic” diagnosis.

We previously demonstrated a strong anti-proliferative and pro-apoptotic effect of PDGFR-alpha signaling, in human and mouse melanoma cells, and human endothelial cells (HUVEC) [30, 31]. In the present study we confirmed the strong anti-proliferative effect in human endothelial and in a larger panel of human melanoma cells, and then addressed in deeper details the mechanisms underlying such anti-proliferation effect. HUVEC and human melanoma (SKMel-28) cells were infected with increasing doses of PDGFR-alpha coding adenovirus (AdCMV.PDGFR-alpha) and control cells were infected with a null virus (AdCMV.null) (10, 30, 100 MOI). Figure 1A and 1B show that PDGFR-alpha overexpression has a marked dose-dependent anti-proliferative effect, reaching 60% and 75% growth inhibition, in HUVEC and SKMel-28 cells, respectively. Figure 1C shows that such biological effect is confirmed in two additional aggressive human melanoma cells, namely A375 and Preyer cells. The overexpression of PDGFR-alpha occurring upon 30 MOI infection dose was then verified at the mRNA level by qRT-PCR (Supplementary Figure S1) and at the protein level (not shown).

### PDGFR-alpha expression in human normal skin biopsies and in melanoma biopsies

Figure 1A–1C demonstrates that PDGFR-alpha overexpression inhibits proliferation in endothelial cells and in at least 3 human melanoma cells lines. We therefore hypothesized that PDGFR-alpha expression should be progressively lost in melanoma as compared to normal skin, as a mechanism to negatively select its inhibitory effect. Such hypothesis was tested by evaluating the expression levels of PDGFR-alpha in 355 human specimens, namely 208 skin melanoma and 147 normal skin, available at IST online (http://ist.medisapiens.com). Figure 1D shows that PDGFR-alpha expression (reported onto the X axis) in melanoma biopsies (black circles) is strongly reduced as compared to normal skin samples (open circles). On the Y axis the expression of beta 2-microglobulin was used as reference. This is consistent with previous evidences showing a clear reduction of PDGFR-alpha expression in melanoma as compared to nevi sections [29]. In Figure 1E a further analysis carried out onto GEO database (http://www.ncbi.nlm.nih.gov/pubmed) indicates that melanoma progression from a primary stage toward a metastatic stage shows a further significant decrease of PDGFR-alpha expression. GDS3966 dataset [49] reports gene-expression profiling in 31 primary melanoma biopsies and in 52 metastatic melanoma biopsies. While the exact amount of neoplastic cells present in the different samples is not available for an accurate normalization, nevertheless mean PDGFR-alpha expression was found to be significantly reduced (p = 0.0002). Immunohistochemistry data available at Human Protein Atlas (http://www.proteinatlas.org/) confirm that PDGFR-alpha expression is undetected in most cancers (http://www.proteinatlas.org/ENSG00000134853-PDGFRA/cancer) while PDGFR-beta is much more expressed (http://www.proteinatlas.org/ENSG00000113721-PDGFRB/cancer). More specifically to melanoma, PDGFR-alpha is undetected in 66% of melanoma tissue sections, while PDGFR-beta is undetected in 43% of melanoma tissue sections.

Data of Figure 1D and 1E support the hypothesis that melanoma onset and progression in vivo selects cells expressing lower levels of PDGFR-alpha.

### Gene expression profiling and validation in endothelial and melanoma cells over-expressing PDGFR-alpha

**Figure 2:**
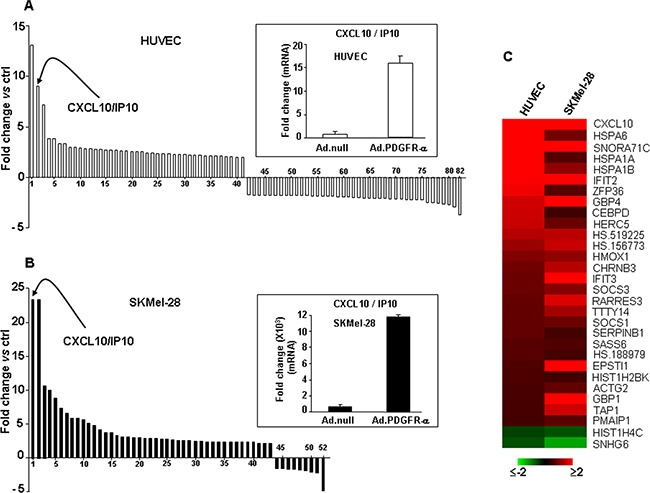
Gene expression profiling and validation in endothelial cells and melanoma overexperessing PDGFR-alpha **A.** Bar graph depicts the top 41 transcripts up-regulated and top 41 down-regulated in HUVEC. Inset reports the corresponding validation by qRT-PCR. Data are reported as mean ± SD of 3 independent experiments. The complete list of 216 differentially modulated transcripts is reported in Supplementary Table S1. **B.** Bar graph depicts the top 43 transcripts up-regulated and top 9 down-regulated in SKMel-28. CXCL10/IP-10 transcript was found strongly up-regulated both in HUVEC and SKMel-28. Inset reports the corresponding validation by qRT-PCR, carried out as 3 independent experiments. Data are reported as mean ± SD. The complete list of 107 differentially modulated transcripts is reported in Supplementary Table S2). **C.** Heatmap depicting all common differentially regulated transcripts in HUVEC and SKMel-28 over-expressing PDGFR-alpha as compared to Ad.null infected cells.

In order to characterize the molecular mechanisms underlying the observed biological effect, gene expression profiling was evaluated in both HUVEC and melanoma cells over-expressing PDGFR-alpha, by Illumina microarray technology. HUVEC and SKMel-28 cells were infected with 30 MOI AdCMV.PDGFR-alpha; the same dose of the AdCMV.null virus was used to infect control cells. A value of at least 1.5 fold increase or decrease combined with a p ≤0.001 was chosen as relevant and significant threshold. In HUVEC overexpressing PDGFR-alpha, the top up- and top down-regulated transcripts (41 in each case) were identified (Figure 2A; the complete list of 216 differentially modulated transcripts is reported in Supplementary Table S1). On the other hand, the top up- and top down-regulated transcripts (43 and 9, respectively) in the SKMel-28 melanoma cells over-expressing PDGFR-alpha are shown in Figure 2B (the complete list of 107 differentially modulated transcripts is reported in Supplementary Table S2). Figure 2A and 2B show that, upon PDGFR-alpha overexpression, a number of genes undergo a similar regulation in HUVEC and in melanoma cells. Namely, CXCL10/IP-10 gene is the most up-regulated in SKMel-28 and the second most up-regulated in HUVEC (23 and 9 fold-increase, indicated by the arrows, respectively). Further, SNORA71C, IFT2 and GBP4 genes are among the most up-regulated in both cells lines (see Supplementary Table S1 and S2). All common differentially expressed transcripts are reported in Figure 2C. Modulation of CXCL10/IP-10 gene-expression reported in Figure 2 was then validated by mRNA quantification in qRT-PCR on an independent set of experiments. Insets of Figure 2A and 2B confirm that PDGFR-alpha overexpression induces a highly significant increase of CXCL10/IP-10 mRNA expressions in both HUVEC (Figure 2A inset) and SKMel-28 melanoma cells (Figure 2B inset). Expression of the unique receptor of CXCL10/IP10 (namely, CXCR3) was found to be not modulated in HUVEC nor in SKMel-28 overexpressing PDGFR-alpha, in both gene expression profiling and qRT-PCR (not shown).

### CXCL10/IP-10 protein expression in endothelial and melanoma cells overexpressing PDGFR-alpha

**Figure 3:**
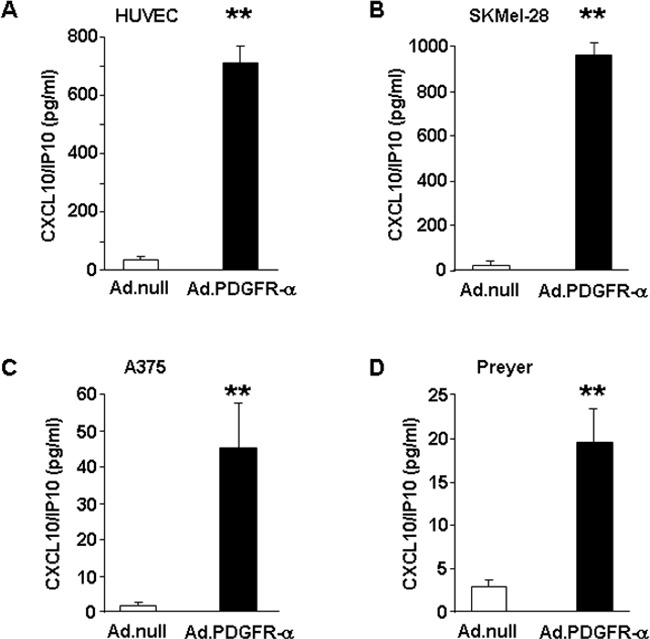
CXCL10/IP10 protein expression CXCL10/IP-10 expression level measured in cell lysates obtained from endothelial (HUVEC) and melanoma (SKMel-28, A375, Preyer) cells over-expressing PDGFR-alpha, as compared to Ad.null-infected cells. Data are reported as mean ± SD of 3 independent experiments and normalized by total protein content.

Since CXCL10/IP-10 was found strongly up-regulated at the mRNA level in both HUVEC and melanoma cells (Figure 2A and 2B), we aimed at validating such modulation at the protein level. Cytokine expression was thus measured in cell lysates obtained from HUVEC and SKMel-28 cells overexpressing PDGFR-alpha. Figure 3A and 3B indicate that CXCL10/IP-10 protein is strongly and significantly up-regulated in PDGFR-alpha-overexpressing cells, as compared to control. A similarly strong and significant effect was then confirmed in two additional human melanoma cells overexpressing PDGFR-alpha, namely A375 and Preyer (Figure 3C and 3D). Such results indicate that transient overexpression of PDGFR-alpha is associated to CXCL10/IP-10 increased expression by several fold vs control, namely, 20 fold in HUVEC, 41 fold in SKMel-28, 103 fold in A375 and 7.6 fold in Preyer cells.

### miRNA expression in melanoma and endothelial cells overexpressing PDGFR-alpha. Profiling experiments and qRT-PCR validation

**Figure 4:**
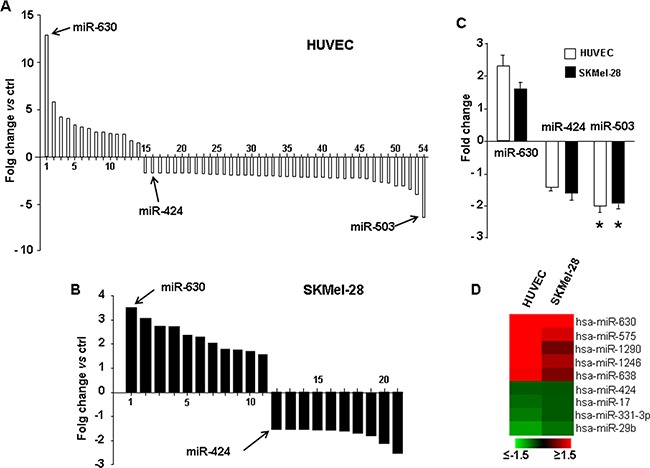
miRNA profiling and qRT-PCR validation miRNome profiling in HUVEC **A.** and SKMel-28 cells **B.** overexpressin PDGFR-alpha vs Ad.null control cells. The complete list of all differentially expressed miRNAs is reported in Supplementary Table S3 and Supplementary Table S4, along with the exact fold changes and computed p values. **C.** qRT-PCR validation, in HUVEC and SKMel-28 cells overexpressing PDGFR-alpha. Data are reported as mean ± SD of 3 independent experiments. **D.** Heatmap depicting all common differentially regulated miRNAs in HUVEC and SKMel-28 over-expressing PDGFR-alpha as compared to Ad.null infected cells.

In addition to gene-expression profiling and protein-level analysis, miRNAs profile in PDGFR-alpha overexpressing cells (HUVEC and SKMel-28) was then analyzed by a miRNA microarray Agilent platform, measuring the expression of over 900 miRNAs. Figure 4A shows that overexpressing PDGFR-alpha leads to the significant up-regulation of 14 miRNAs in HUVEC. The most up-regulated is miR-630 (more than 12 fold, p = 0.0006 vs control). On the other hand, 39 miRNAs were found to be significantly reduced; the most down-regulated is miR-503 (6.4 fold reduction, p = 0.016 vs control). Interestingly enough, miR-424, another member of the miR-503 cluster, was also found down-regulated about 2 fold. In SKMel-28 cells, the overexpression of PDGFR-alpha leads to the significant up-regulation of 11 miRNAs and reduction of 10 miRNAs (Figure 4C). Nine miRNAs resulted to be commonly differentially expressed between HUVEC and SKMel-28 cells as reported in the heatmap (Figure 4D). Validation is reported in Figure 4C. qRT-PCR analysis on an independent set of HUVEC and SKMEL-28 cells over-expressing PDGFR-alpha was carried out. miR-503 was confirmed to be strongly and significantly down-regulated both in HUVEC and in SKMel-28 (fold-change -2 with p = 0.0008 and = 0.004, respectively). The up-regulation of miR-630 and the down-regulation of miR-424 reported in Figure 4A were also confirmed by qRT-PCR in both HUVEC and SKMel-28 (Figure 4B). These data indicate that miR-503 is strongly down-regulated in both HUVEC and SKMEL-28 cells overexpressing PDGFR-alpha, as compared to Ad.CMV.null control cells. All differentially expressed miRNAs are reported in Supplementary Table S3 and Supplementary Table S4, along with the exact fold changes and computed p values.

### mir-503 target prediction and target validation

**Figure 5:**
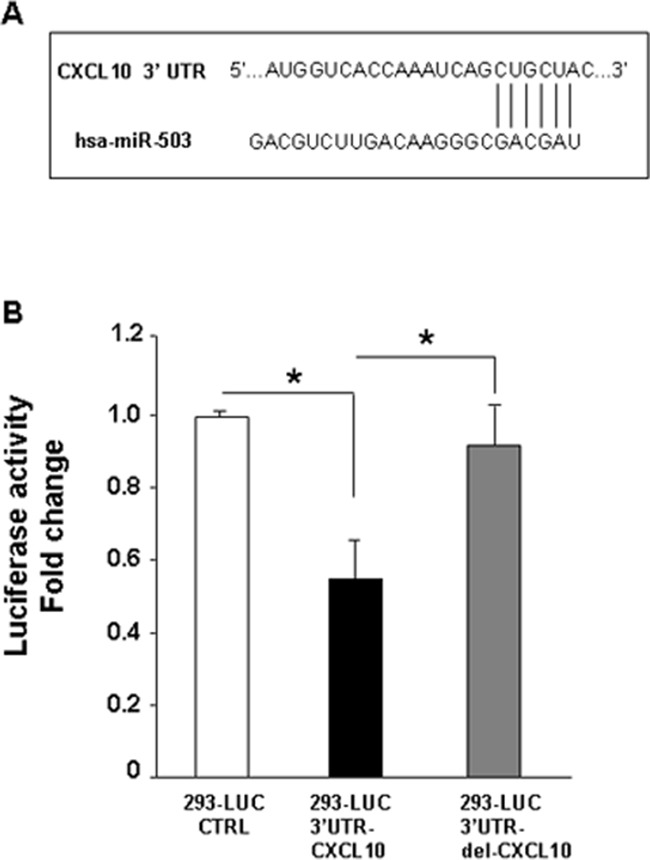
Target prediction of miR-503 and validation **A.** CXCL10/IP-10 was predicted to be one of the miR-503 targets according to TargetScan software, given the complementarity of CXCL10/IP-10 3′UTR and miR-503 seed sequence. **B.** 3′-UTR-Luc assay shows a significant decrease (about 50%, *p value* < 0.0001) in Luciferase reporter expression in transiently transfected 3′-UTR CXCL10/IP10 Luciferase stable 293 cell line with miR-503, as compared to the control vector, and a significant reversion is observed when a deleted form of the 3′-UTR CXCL10/IP-10 was used as specificity control. Data are reported as mean ± SD of 3 independent experiments.

Figure 2 and Figure 3 show the relevant increased expression of CXCL10/IP-10 at both mRNA and protein levels, as result of PDGFR-alpha overexpression. Figure 4A and 4B show the strong reduction miR-503 expression in the same experimental set up. Therefore we hypothesized a functional interplay between CXCL10/IP-10 and miR-503, downstream the PDGFR-alpha. According to TargetScan software, CXCL10/IP-10 is one of the predicted miR-503 targets, given the computed high score of complementarity of CXCL10/IP-10 3′UTR and miR-503 seed sequence (Figure 5A). To experimentally confirm such prediction a 3′-UTR-Luc assay was carried out. A transient transfection of 3′-UTR CXCL10/IP-10 Luciferase stable 293 Cell Line with miR-503 led to a significant decrease (about 50%, p < 0.0001) in Luciferase reporter expression as compared to the control vector (Figure 5B). Such effect was significantly reverted when a deleted form of the 3′-UTR CXCL10/IP-10 was used as specificity control (Figure 5B), demonstrating that the 3′-UTR CXCL10/IP-10 is a functional target of miR-503, as predicted by TargetScan.

### CXCL10/IP-10 neutralization reverts PDGFR-alpha-dependent growth inhibition and dasatinb reverts PDGFR-alpha dependent overexpression of CXCL10/IP10

**Figure 6:**
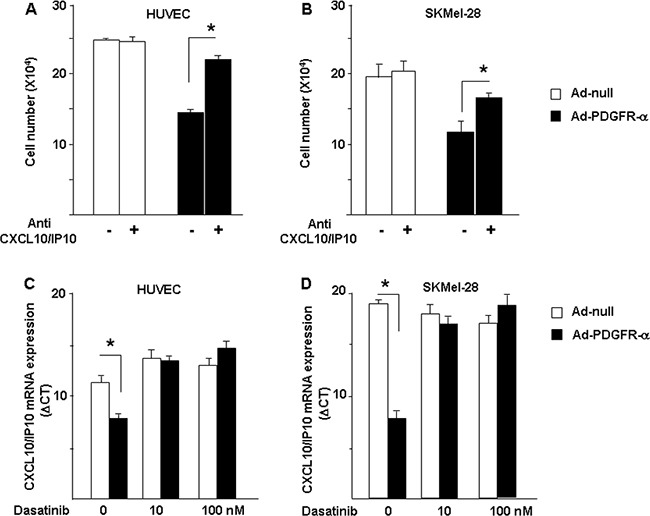
Mechanisms characterization Neutralization of CXCL10/IP10 reverts PDGFR-alpha-dependent growth inhibition. The key role of CXCL10/IP-10 to mediate the anti-proliferation effect of PDGFR-alpha was confirmed by the use of the neutralizing antibody anti-CXCL10/IP-10, which partially but significantly reverts the inhibitory effect of PDGR-alpha overexpression, in both HUVEC **A.** and SKMel-28 **B.** cells. The anti-tumor drug Dasatinb, known inhibitor of PDGFR-alpha and other tyrosine-kinases, completely restores CXCL10/IP10 expression (expressed as ΔCT). Data are reported as mean ± SD of 3 independent experiments.

Figures 2 to 5 represent strong evidences allowing us to hypothesize that CXCL10/IP-10 and miR-503 are involved in the PDGFR-alpha anti-proliferation activity shown in Figure 1. Cells overexpressing PDGFR-alpha were then exposed to the specific neutralization of CXCL10/IP-10. As reported in Figure 6A and 6B, treatment with the neutralizing CXCL10/IP-10 antibody significantly reverted the growth inhibition given by PDFR-alpha overexpression, in both HUVEC and SKMel-28 cells. Such results allowed us to conclude that CXCL10/IP-10 represents a key player of the anti-proliferation action of PDGFR-alpha. To further characterize the observed effects, we used an antitumor drug currently in clinical practice, namely Dasatinib. It is a known inhibitor of PDGFR-alpha and other tyrosin kinase receptors. Under Dasatinb treatment, the CXCl10/IP10 upregulation induced by PDGFR-alpha was completely reverted, further suggesting that the observed modulation of CXCL10/IP10 is PDGFR-alpha related.

### Integrated omics analysis

An exhaustive bio-informatic analysis was then carried out to integrate the obtained transcriptomics and miRNomics data. According to the stringent criteria reported in Materials and Methods section, genes and miRNAs up- and down-regulated vs the corresponding controls, in cells over-expressing PDGFR-alpha, were analyzed to predict the affected molecular pathways. Such analysis was carried out with two different bio-informatics approaches: i.e, via KEGG pathways analysis and via Ingenuity Pathway Analysis (IPA) software. KEGG analysis showed that HUVEC over-expressing PDGFR-alpha undergo the significant up-regulation of 13 molecular pathways, and the significant down-regulation of 5 molecular pathways, listed in Table 1. On the other hand, KEGG analysis revealed that SKMel-28 cells over-expressing PDGFR-alpha undergo the significant up-regulation of 5 molecular pathways (4 of which being upregulated also in HUVEC), while no pathways were found to be significantly down-regulated. The Cytokine-Cytokine receptor interaction pathway was the most strongly affected both in HUVEC and in SKMel-28 cells. In fact, the expression of 9 genes and 6 genes of this pathway was modified in HUVEC and SKMel-28 in a significant manner (p =1 × 10-11 and p = 6 × 10-7 respectively) (see Table 1).

**Table 1:** KEGG signaling pathways significantly affected by PDGFR-alpha over-expression, in HUVEC and in SKMel-28

KEGG pathways					
*UP REGULATED IN HUVEC*					
		N. of genes found to be regulated	% of all genes present in the pathway	t test p value	Benjamini p value
Cytokine-cytokine receptor interaction	*	9	8.6	1 E-11	8 E-10
Pathways in cancer		7	6.7	6 E-7	2 E-5
NOD-like receptor		5	4.8	2 E-6	6 E-5
Jak-STAT signaling	*	5	4.8	5 E-6	8 E-5
Chemokine signaling pathway	*	5	4.8	2 E-5	3 E-4
Toll-like receptor		4	3.8	1 E-4	0.01
MAPK signaling pathway		5	4.8	1 E-4	0.01
TGF-beta signaling pathway		3	2.9	0.002	0.01
Bladder cancer		3	2.9	0.002	0.01
Cell adhesion molecules (CAMs)		3	2.9	0.002	0.01
Neuroactive ligand-receptor interaction		3	2.9	0.002	0.01
Antigen processing and presentation	*	3	2.9	0.003	0.02
Cell cycle		3	2.9	0.007	0.04
***DOWN REGULATED IN HUVEC***					
Oocyte meiosis		3	3.6	5 E-4	0.002
Ubiquitin mediated proteolysis		3	3.6	0.002	0.005
Endocytosis		3	3.6	0.003	0.004
Cell cycle		3	3.6	0.003	0.004
Axon guidance		3	3.6	0.006	0.007
***UP REGULATED IN SKMel-28***					
Cytokine-cytokine receptor interaction	*	6	7	6 E-7	2.00E-05
Chemokine signaling pathway	*	5	5.8	8.00E-06	1.00E-04
RIG-I-like receptor signaling pathway		3	3.5	7.00E-03	0.09
Jak-STAT signaling	*	3	3.5	0.007	0.09
Antigen processing and presentation	*	3	3.5	0.01	0.09
***DOWN REGULATED IN SKMel-28***					
		No pathway			

* Pathways upregulated both in HUVEC and SKMel-28

**Table 2:** Primers used in the qRT-PCR analysis

human PDGFR-alpha Fw	5′-tttgatttcttccagcattgtg-3′
human PDGFR-alpha Rev	3′-aggtggttgaccttcaatgg-5′
human CXCL10/IP10 Fw	5′-gacatatactccatgtagggaagtga-3′
human CXCL10/IP10 Rev	3′-gaaagcagttagcaaggaaaggt-5′
human beta 2-microglobulin Fw	5′-ttctggcctggaggctatc-3′
human beta 2-microglobulin Rev	3′-tcaggaaatttgactttccattc-5′
human PDGFR-beta Fw	5′-gagacgttgatggatgacacc-3′
human PDGFR-beta Rev	5′-catctgcaaaaccaccattg-3′
human CXCR3 Fw	5′-caaccacaagcaccaaagc-3′
human CXCR-3 Rev	5′-tcttctgcgtgatcccatc-3′

**Figure 7:**
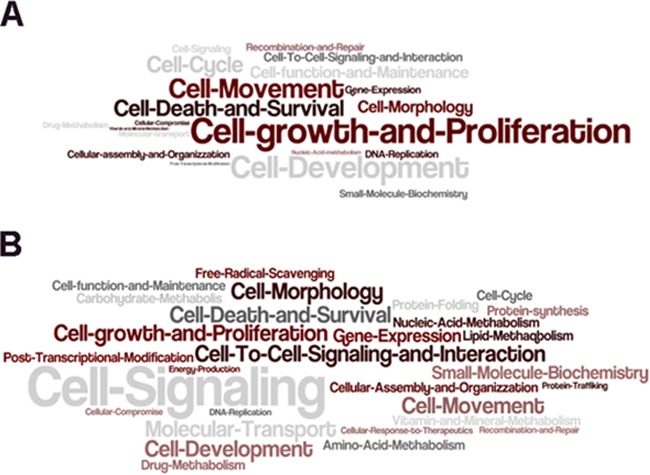
IPA Functional Analysis Word Cloud analysis of significantly enriched molecular functions identified by IPA functional analysis considering differentially expressed transcripts in HUVEC **A.** and SKMel-28 cell **B.** over-expressing PDGFR-alpha. The text size is inversely proportional to the log *p value* ≤ 0.05 (Fisher's exact test).

An additional approach was carried out with the IPA analysis, which highlighted the relevant effect of PDGFR-alpha overexpression on several molecular functions, including Cell Signaling, Cell Growth and Proliferation, Cell Development, Cell Movement, Cell Death and Survival, confirming the KEGG analysis results. The molecular functions significantly affected in HUVEC and SKMel-28 cells are reported in Word Cloud (Figure 7A and 7B, respectively). Supplementary Table S5A and S5B report the differentially expressed transcripts and the predicted affected Top Diseases and Functions by network they form, in HUVEC and SKMel-28, respectively, according to IPA functional annotation. Several Top Diseases and Functions are commonly affected in the two cell systems analyzed, such as Cancer, Inflammatory *response*, *Hematological System Development and Function*, *Cell to Cell Signaling and Interaction*, *Tissue morphology*, *Cell cycle*, *Cell Death and Survival*, *Cardiovascular Disease*. The Top Diseases and Functions identified with the highest score comprise *Cancer*, *Cell Death and Survival* and *Inflammatory Response*.

Data from Figure 2A and Figure 4 were further integrated. Namely, within the list of deregulated transcripts reported in Figure 2A, IPA software identified the highly predicted or experimentally validated targets of the miRNAs differentially expressed reported in Figure 4. Supplementary Table S6 reports the whole list of differentially expressed mRNAs targeted by differentially expressed miRNAs, in addition to CXCL10/IP-10. The miR-503/CXCL10/IP-10 represents the best match showing the highest miRNA down-regulation (namely, -6.39 fold decrease) and the highest mRNA transcript up-regulation (+9 fold increase).

**Figure 8:**
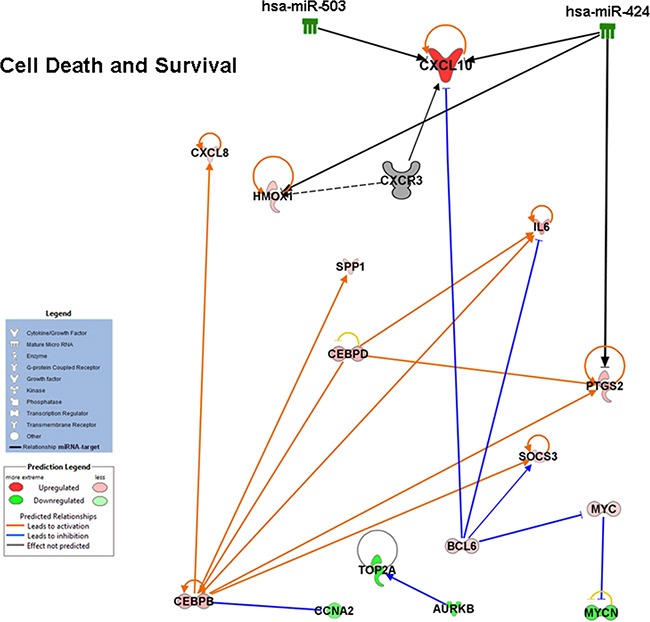
Integration of miRNA and transcriptome changes occurring in HUVEC cells Functional network obtained by IPA analysis highlighting inverse relationships between miRNA and transcripts expression induced by PDGR-alpha overexpression. Gene expression- and miRNA- microarray data were integrated.

The molecular pathways putatively affected by the miRNAs deregulation were then also predicted in PDGFR-alpha overexpressing HUVEC. As an example, Figure 8 shows that several transcripts found to be up- or down-regulated fall within the “Cell death and Survival” functional category. This Figure represents a narrow snapshot of the complex molecular status observed in cells overexpressing PDGFR-alpha. It shows the reduced levels of miR-503 and miR-424, the increased level of CXCL10/IP-10, and the observed up- and down- regulation of other factors. The arrows report the known functional connections, according to IPA software database. Most interestingly, CEBPB and CEBPD transcriptor factors are found up-regulated. CEBPB shares functional interactions with CXCL10/IP-10 [50], and both CEBPB and CEBPD are known to be tumor suppressors [51–54]. Therefore their up-regulation in HUVEC overexpressing PDFR-alpha may explain, at least in part, the observed anti-proliferation effect.

## DISCUSSION

We have previously shown a direct molecular and functional interplay between FGF-2 and PDGF family members, by demonstrating that such ligands interact with high affinity, inducing hetero-dimerization of the corresponding receptors [30], leading to a relevant inhibition of cell growth in both endothelial [55] and melanoma cells, either *in vitro* and *in vivo* mouse melanoma models [56]. Such inhibitory role of PDGFR-alpha appears not totally surprising, given that the other main receptor of this family, PDGFR-beta, is highly related to the v-SIS oncogene of simian sarcoma virus. While PDGFR-alpha has been recently investigated in melanoma surrounding fibroblast [44], in the current study we addressed PDGFR-alpha action in other key players within the melanoma tissue, i.e., endothelial and melanoma cells. Overexpressing PDGFR-alpha allowed us to force the signaling toward a PDGFR-alpha/alpha signaling and to follow biological and molecular effects mostly related to a pure PDGFR-alpha/alpha signal. Under such conditions, a clear anti-proliferation action in both endothelial and melanoma cells has been found, consistently with previous reports [29, 31, 32].

The inhibitory role of PDGFR-alpha in melanoma was supported by data collected in several hundred melanoma patients' biopsies, showing a significant reduction of PDGFR-alpha expression in melanoma biopsies as compared to normal skin, as well as in metastatic melanoma biopsies as compared to primary melanoma biopsies (Figure 1D, 1E and datasets GDS1375, GDS1989, GDS2200, GDS1965 available at GEO database site http://www.ncbi.nlm.nih.gov/sites/entrez) [31]. This indicates that PDGFR-alpha is lost in melanoma tissue, likely due to its inhibitory effect. A negative selection process may therefore occur, thus supporting the hypothesis that melanoma progression *in vivo* may select cells expressing low or very-low levels of PDGFR-alpha.

The current study shows that overexpression of PDGFR-alpha is associated with a strong induction of the pro-inflammatory cytokine CXCL10/IP-10 (Figure 2 and Figure 3). Inflammation appears as one of the most affected function in cells over-expressing PDGFR-alpha (Supplementary Table S5A and S5B). This finding is consistent with previous data showing that the onset of an appropriate inflammatory response may finally exert anti-tumor activity [57]. Figure 8 shows the functional interconnections of different pro-inflammatory factors up-regulated in PDGFR-alpha over-expressing cells, such as IL6 and CXCL8, further suggesting the hypothesis that an inflammatory response may be involved in the observed anti-angiogenesis and anti-melanoma effect of PDGFR-alpha. Interestingly, elsewhere an anti-angiogenic and anti-melanoma effect has been reported to be related to a pro-inflammatory state mediated by pro-inflammatory cytokines such as CXCL10/IP-10 [58]. We then conclude that the activation of pro-inflammatory pathways may underlie, at least to a certain extent, the anti-angiogenic and anti-melanoma effect of PDGFR-alpha via CXCL10/IP-10.

The protective role of CXCL10/IP-10 has been already investigated in melanoma [23, 27, 59–61]. The present study for the first time unreveals direct connection with the PDGF-R alpha signalling. In facts, the neutralizing anti-CXCL10/IP-10 antibody was found to revert the anti-proliferation effect in cells overexpressing PDGFR-alpha (Figure 6) further confirming the effects shown in Figures 2 and 3. An additional support was found *in vivo* in 353 human biopsies samples from http://ist.medisapiens.com/ (Figure 1D). Such data indicate that PDGFR-alpha and CXCL10/IP-10 expression levels show a significant negative correlation (R= − 0.2) in healthy skin while it turns to a significant positive correlation (R = + 0.2) in melanoma human specimens (p < 0.001 in either cases). Finally, when the correlation index was computed on melanoma data reported in Figure 1E, PDGFR-alpha expression levels show a significant positive correlation with CXCL10/IP10 levels (p = 0.002) while PDGFR-beta levels show no significant correlation (p = 0.6), further supporting a specific functional link of PDGFR-alpha with CXCL10/IP10.

We therefore hypothesized that the biological effects of PDGFR-alpha may be mediated, at least in part, by CXCL10/IP-10. This cytokine is known to be directly involved in two molecular pathways, namely Cytokine-Cytokine receptor interaction, and Toll-like receptor signaling pathway, according to the CXCL10 Cancer GeneticsWeb card (see http://www.cancerindex.org/geneweb/CXCL10.htm). Both these pathways were predicted to be significantly affected in cells over-expressing PDGFR-alpha, as compared to control cells. This was observed in HUVEC over-expressing PDGFR-alpha (see Table 2) and the first pathway was found to be affected also in SKMel-28 overexpressing PDGFR-alpha (see Table 2), confirming that PDGFR-alpha-related- may overlap CXCL10/IP-10- related-pathways, including the inflammation-related ones.

Overexpressing PDGFR-alpha has been found in the present study to significantly reduce miR-503 expression. Since we also demonstrate that CXCL10/IP-10 is a validated miR-503 target, down-regulation of this miRNA may explain, at least in part, the observed CXCL10/IP-10 up-regulation. In the recent few years miR-503 has been found to exert opposite effects in different cancer settings; namely it shows a protective role by inhibiting proliferation and inducing apoptosis in glioma [62] and in prostate cancer [63], while on the contrary its down-regulation has shown a protective role in esophagus carcinoma [64] and osteosarcoma [65]. Additional data indicate contrasting effect of miR-503 in colon carcinoma [66, 67].

We also found a significant down-regulation of miR-424 both in HUVEC and melanoma cells overexpressing PDGF-R alpha. miR-424 has been implicated in angiogenesis regulation [68, 69], and suppression of the miR-424-503 gene has been shown to promote mucosal defence in bacterial infection via CX3CL1 expression [70].

Further, we observed that PDGFR-alpha over-expression strongly induces miR-630 expression. This miRNA is known to promote apoptosis and autophagy in cancer cells [71–74]; itis a key negative regulator incancer progression [75] and may represent a further mechanism underlying the anti-proliferation activity of PDGFR-alpha.

**Figure 9:**
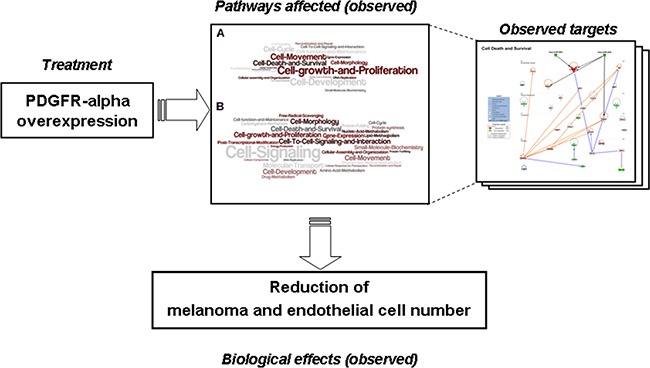
Cartoon summarizing the intervention and the cellular reactions observed in the current study Over-expressing PDGFR-alpha induces reduction of melanoma and endothelial cell number affecting several molecular functions via up- and down- regulation of several interconnected molecules.

In conclusion, the cartoon reported in Figure 9 summarizes the interventions and the cellular reactions observed in the present study. As depicted, over-expressing PDGFR-alpha reduces melanoma and endothelial cell number by affecting several molecular functions (such as *Cell-growth and proliferation*; *Cell Death and Survival*, *Cell Signaling*) via up- and down- regulation of several interconnected molecules. Namely, CXCL10/IP-10, miRNA-503, CEBPB and CEBPD, two known onco-suppressor genes [50, 51, 53, 54, 52] were found strongly modified in cells over-expressing PDGFR-alpha, along with other molecules such as CXCL8, IL6, SOCS3, all known to have functional connections with CXCL10/IP-10.

The current study is the first indicating that the anti-melanoma role of PDGFR-alpha may be linked to the known anti-melanoma role of CXCL10/IP-10, and suggests PDGFR-alpha and CXCL10/IP-10 as related targets in melanoma therapy, likely via miR-503.

## MATERIALS AND METHODS

### Cell culture

Human umbilical vein endothelial cells (HUVEC; Clonetics, Lonza) were maintained in complete medium containing endothelial cell basal medium (EBM-2; Clonetics, Lonza) supplemented with endothelial cell Bullet Kit (2% FCS, hEGF-2, hFGF-2, hVEGF, R3-IGF-1, ascorbic acid, hydrocortisone, heparin, gentamicin, amphotericin-B; Clonetics, Lonza). Cells were used for the experiments between passage 3rd and 5th. SKMel-28 (ATCC), A375 (ATCC) and Preyer cells (kindly provided by Dr. Tobias Haas, ISS-Rome), were maintained in complete medium Dulbecco's modified Eagle's medium (DMEM; Hyclone, South Logan, UT) supplemented with 10% FBS (HyClone), 2 mM L-glutamine and 100 IU/ml penicillin/streptomycin (Invitrogen, Carlsbad, CA) in humidified 5% CO_2_ atmosphere, at 37 °C as described [76]. The culture medium was changed every 3 days and when cells were sub-confluent;monolayers were harvested by 1 minute exposure to 0.1% Trypsin-EDTA (Life Technologies Inc).

### Construction of PDGFR-alpha adenovirus vector

The recombinant adenoviral vector expressing EGFP and human PDGFR-alpha (AdCMV.PDGFR-alpha) and the null (AdCMV.null) control were generated by homologous recombination in bacteria according to a standard procedure [77] as previously reported [31].

### Proliferation assay in endothelial- and melanoma cells over-expressing PDGFR-alpha

HUVEC and SKMel-28 cells (5×10^5^ cells in 100 mm dish) were infected for 2 hours with AdCMV.PDGFR-alpha or with AdCMV.null (10, 30, 100 Multiplicity Of Infection - MOI) in serum-free medium. Cells were then recovered in complete fresh medium and cultured for additional 48 hours. Then cells were harvested and counted with a hemacytometer and total RNA or protein extracts were prepared as reported below as described [78]. The infection-efficiency reached at least 65% in all cases. For neutralizing antibody experiments: HUVEC and SKMel-28 cells were infected with 30 MOI of AdCMV.PDGFR-alpha or with AdCMV.null for 2 hours, recovered in complete fresh medium and treated with the anti-human CXCL10/IP-10 neutralizing antibody (3 μg/ml; R&D Systems, Minneapolis, MN) for 48h. Cells were then harvested and counted as described above. Dasatinib was from Sigma-Aldrich.

### PDGFR-alpha expression *in vivo* by *in silico* analysis

IST online is a database reporting gene-expression data from thousands patients of several cancer types, available at IST online Medisapiens (http://www.medisapiens.com/). The platform reportscorrelation dot-plots graphs referring to the expression of two genes selected by the operator, indicated in the X and Y axes. We investigated PDGFR-alpha gene expression *vs* the beta 2-microglobulin (taken as a housekeeping gene) in 355 human samples (208 skin melanoma and 147 normal skin). Additional *in silico* analyses were carried out by accessing the GEO database at (http://www.ncbi.nlm.nih.gov/pubmed) on the GDS3966 dataset, by analysing PDGFR-alpha expression in primary versus metastatic melanoma human biopsies in 83 patients (31 primary melanoma and 52 metastatic melanoma) (analysis carried out on GDS3966/ 203131_at / PDGFRA).

### RNA extraction

RNA extraction from infected cells was obtained as follows: after medium removal, cells were treated with TRIzol reagent (Invitrogen Corporation, Carlsbad, California, USA). Total RNA was isolated from the samples as described earlier [79]. Before use, the RNA concentration in each sample was assayed with NanoDrop 2000C spectrophotometer (NanoDrop, Thermo Scientific, Rockford, IL) and its quality was assessed with an Agilent 2100 Bioanalyzer with the Agilent RNA 6000 nano kit (Agilent Technologies, Santa Clara, CA) as previously described [80, 81].

### Quantitative Real-time PCR (qRT-PCR)

According to standard procedures, cDNA was synthesized with Superscript III (Invitrogen-Life Technologies Italia, Monza, Italy) and mRNA expression was analyzed using the SYBR-Green qRT-PCR method (5 ng/assay) (Qiagen, Hilden, Germany) according to the manufacturer's instructions. Quantification was achieved with ABI Prism 7000 SDS (Applied Biosystems, Monza, Italy). mRNA expression value was then normalized for beta 2-microglobulin levels.

Table 2 reports the primers used to this aim. Micro-RNA (miRNA) levels were analyzed using the Applied Biosystems TaqMan quantitative qRT-PCR method (1 ng/assay) performed according to the manufacturer's instructions and quantified with the ABI Prism 7000 SDS (Applied Biosystems, Monza, Italy). Mature miRNA levels were normalized to miR-16, whose expression is constant in all tested RNA samples. For both mRNAs and miRNAs, relative expression was calculated using the comparative Ct method (2-ΔΔCt).

### Gene expression microarray and analysis

Three independent biological replicates for each condition (HUVEC cells overexpressing or not-overexpressing PDGFR-alpha, and SKMel-28 cells overexpressing or not-overexpressing PDGFR-alpha) were pooled to obtain 4 samples of 500 ng of total RNA as starting material for the synthesis of cDNA and biotinylated cRNA, according to the Illumina TotalPrep RNA Amplification Kit protocol (Ambion, Austin, TX, USA). Then, for each sample, 750 ng of cRNA were hybridized in triplicate on Illumina HumanHT-12 v4.0 BeadChips (Illumina Inc., USA) as already described [82] and subsequently scanned with the Illumina iSCAN. Data analyses were performed with GenomeStudio software version 2011.1 (Illumina Inc., USA) as reported in the Statistics section of the Methods. Raw microarray data have been deposited, in a format complying with the Minimum Information about Microarray Gene Experiment guidelines of the Microarray Gene Expression Data Society, in the EBI Array-Express database (www.ebi.ac.uk/arrayexpress) with accession number E-MTAB-4261.

### Cytokine quantification

Fifty microliters of HUVEC and SKMel-28 lysates (0.8 μg total protein/μl) were analyzed to measure cytokine expression with the Bio-Plex Cytokines assay kit (BioRad Laboratories, Milan, Italy), a magnetic-bead based immunoassay. Sample dilution (1:4 in the sample-dilution buffer) and handling were carried out strictly according to manufacturer's instructions. After incubation with antibodies-activated magnetic beads, samples were washed using a Bio-Plex ProTM Station (Bio-Rad). The quantification was carried out on a Bio-Plex® 200 System (Bio-Rad) run by a Bio-Plex Manager Software version 6.1 and results were expressed as pg/ml. Normalization of samples was achieved by correcting for total protein concentration of cell lysates. Data are expressed as mean ± standard deviation (SD). Three independent experiments were carried out.

### miRNA microarray and data analysis

For miRNA expression profiling, three independent biological replicates for each condition (i.e., HUVEC and SKMel-28 cells overexpressing or not PDGFR-alpha) were used and pooled to obtain 4 samples. For each sample, total RNA (800 ng) was used as input for labeling reaction and hybridization according to the miRNA Microarray System with miRNA Complete Labeling and Hyb Kit protocol (Agilent Technologies). Labeled RNAs were then hybridized in technical triplicate on Agilent human miRNA microarray rel. 14, V2, 8×15K (Agilent Technologies) and acquired by using Agilent Microarray Scanner G2565BA (Agilent Technologies). Data analysis was performed with Agilent Feature Extraction v.10.7.3.1, Gene spring v.11.5 (Agilent Technologies). Data were analyzed as reported in Statistics section of Methods. Raw miRNA microarray data for HUVEC and SKMel-28 cells have been deposited, in a format complying with the Minimum Information about Microarray Gene Experiment guidelines of the Microarray Gene Expression Data Society, in the EBI Array-Express database (www.ebi.ac.uk/arrayexpress) with accession number E-MTAB-4266.

### Luciferase reporter assay

CXCL10/IP-10 3′UTR Luciferase Stable 293 Cell Line (purchased from abm) containing target site sequences complementary to the seed sequence of miR-503 cloned downstream of the luciferase gene was used. Moreover, 27 nucleotides deleted sequence (5′-tgatggtcaccaaatcagctgctacta -3′), at 541 to 567 3′UTR of human CXCL10/IP-10 Luciferase Stable 293 Cell Line and Blank (Control) 3′UTR Luciferase Stable 293 Cell Line were also used (abm). For the reporter assay, cells were transiently transfected with 800 ng pEP-miR-503 or with pEP-miR-Null Control (Cell Biolabs, inc.) using FuGENE 6 Transfection Reagent (Roche). Each sample was co-transfected with 80 ng of pRL-TK plasmid expressing Renilla luciferase (Promega, Madison). Cell collection was carried out 48 h after transfection and analyzed using the Dual-Luciferase Reporter Assay System (Promega, Madison). Relative luciferase activity was normalized to Renilla luciferase activity. Transfections were performed in triplicate and repeated at least 3 times in independent experiments.

### Bioinformatic analysis of genes and miRNAs expression profiling

The list of genes differentially expressed has been analyzed for Gene Ontology terms enrichment and KEGG pathway involvement by means of DAVID v. 6.7 online tool (Database for Annotation, Visualization and Integrated Discovery, at https://david.ncifcrf.gov/). Lists of up- and down-regulated genes were analyzed with reference to background list consisting of the detected genes in the same experimental conditions in control cells. For miR-503, putative mRNA targets prediction was carried out using TargetScan software [83–86].

TargetScan provides computationally predicted miRNA gene targets by searching for the presence of 8-mer and 7-mer sites matching the seed region of each miRNA.

The list of modified transcripts was also analyzed using Ingenuity Pathway Analysis Software (IPA, Ingenuity® Systems, www.ingenuity.com). It refers to a proprietary knowledge base (Ingenuity Pathways Knowledge Base) where biological interactions and functional properties are annotated. IPA Functional Analysis on “molecular and cellular functions” category and Canonical Pathway investigation were carried out calculating the likelihood that the association between a given transcription dataset and a specific function or pathway is due to random choice, and it is expressed as a *p value* calculated using the right-tailed Fisher Exact Test. For network generation, each differentially expressed transcript identifier was uploaded and mapped to its corresponding object in Ingenuity Knowledge Base to algorithmically generate molecular networks based on their connectivity. Obtained networks are scored according to a numerical value (Score) taking into account the number of dataset molecules they contain and the network size as well as the total number of input transcripts in the dataset and the total number of molecules in the Ingenuity Knowledge Base that could potentially be included in the networks. The network Score is based on the hypergeometric distribution and is calculated with the right-tailed Fisher Exact Test. Finally, the “microRNA Target Filter” tool was used to provide insights into the biological effects of microRNAs, using experimentally validated interactions from TarBase and miRecords, as well as predicted microRNA-mRNA interactions from TargetScan examining microRNA-mRNA pairings in the pathways of interest.

### Statistical analysis

Proliferation data, qRT-PCR data, cytokine expression and luciferase activity data were analyzed with GraphPad Prism 4.0 software packages (Graph Pad Inc. San Diego, CA). A statistical significance threshold of *p* < 0.05 was considered. Normal distribution of datasets was tested according to D'Agostino and Pearson omnibus normality test. A 2 tails unpaired t Test assay was then performed if normality test was passed. A non-parametric Mann-Whitney test was carried out if D'Agostino and Pearson normality test was not passed. Benjamini test was carried to identify pathways significantly affected in over-expressing cells. For gene expression analysis, data were normalized with the quantile algorithm, and genes were considered detected if the detection *p-value* was less than 0.05. Statistical significance was calculated with Illumina DiffScore, a proprietary algorithm that uses the bead standard deviation to build an error model. Only genes with at least 1.5 fold increase or decrease and DiffScore of ≤-30 or ≥30, corresponding to a *p-value* cut off ≤0.001, were considered as statistically significant by comparing all values obtained in HUVEC cells overexpressing PDGFR-alpha *vs* HUVEC control cells as well as SKMel-28 cells overexpressing PDGFR-alpha *vs* SKMel-28 control cells.

For miRNA microarray data analysis, a quantile-normalization was applied and statistical significance was calculated by using the unpaired t-Test for two groups. Only miRNAs with at least 1.5 fold increase or decrease and *p value* cut-off ≤0.05, were considered as differentially expressed with statistical significance, by comparing all values obtained in HUVEC cells overexpressing PDGFR-alpha vs HUVEC control cells as well as SKMel-28 cells overexpressing PDGFR-alpha vs SKMel-28 control cells.

## SUPPLEMENTARY MATERIALS FIGURES AND TABLES














