# Supplementary material for: PDGFR-alpha inhibits melanoma growth via CXCL10/IP-10: a multi-omics approach
Source: Oncotarget. 2016 Oct 13;7(47):77257–75. doi: 10.18632/oncotarget.12629 (PMC5363585; doi:10.18632/oncotarget.12629)
Supplement: Supplementary file 1 [file oncotarget-07-77257-s001.pdf]

PDGFR-alpha inhibits melanoma growth via CXCL10/IP-10: a multi-*omics* approach

SUPPLEMENTARY FIGURES AND TABLES

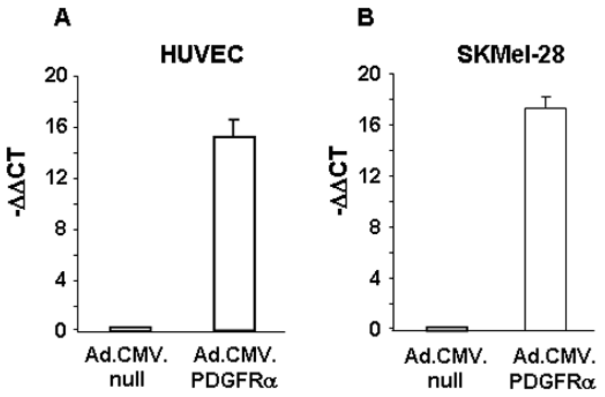

Supplementary Figure S1: qRT-PCR analysis shows the strong overexpression of PDGFR-alpha mRNA in HUVEC (N = 6) and SKMel-28 (N = 6) (A and B respectively) upon 30 MOI infection dose with AdCMV. PDGFR-alpha (48 hours).

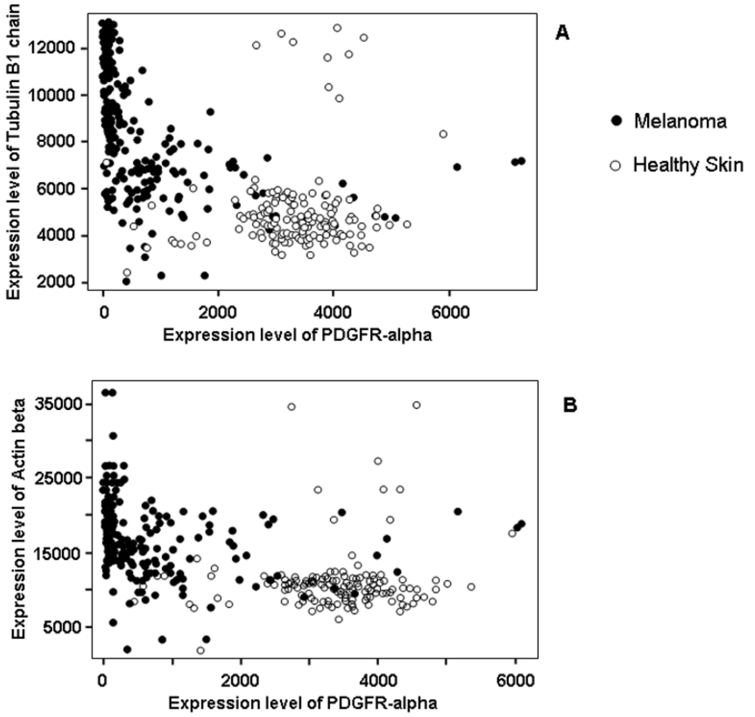

Supplementary Figure 2SA-2SB: reports correlation plots of PDGFR-alpha with housekeeping genes other than beta-2 microglobulin, namely Tubulin Beta 1 gene (S2A) and Actin beta gene (S2B). In all cases PDGFR-alpha is strongly reduced in melanoma samples vs normal skin samples.

Supplementary Table S1: Differentially expressed transcripts in HUVEC cells overexpressing PDGFR-alpha *vs* Ad.null ctrl cells (216 genes). In bold the transcripts reported in Figure 2A.

See Supplementary File 1

Supplementary Table S2: Differentially expressed transcripts in SKMel-28 cells overexpressing PDGFR alpha vs Ad.null ctrl cells (107 genes). In bold the transcripts reported in Figure 2B.

See Supplementary File 2

Supplementary Table S3: Differentially expressed miRNAs in HUVEC cells overexpressing PDGFR-alpha *vs* Ad.null ctrl cells.

See Supplementary File 3

Supplementary Table S4: Differentially expressed miRNAs in SKMe-l28 cells overexpressing PDGFR-alpha vs Ad.null ctrl cells

miRNA	Fold-change	p-value
hsa-miR-630	3.48	0.00198
hsa-miR-1977_v14.0	3.03	0.02592
hsa-miR-135a*	2.70	0.04333
hsa-miR-150*	2.68	0.00065
hsa-miR-762	2.33	0.01027
hsa-miR-575	2.27	0.01446
hsa-miR-1246	2.00	0.01079
hsa-miR-638	1.75	0.00562
hsa-miR-1181	1.72	0.03901
hsa-miR-1290	1.67	0.01835
hsa-miR-1915	1.53	0.04932
hsa-miR-424	-1.52	0.03304
hsa-miR-331-3p	-1.53	0.04243
hsa-miR-17	-1.53	0.04787
hsa-miR-129-3p	-1.54	0.02699
hsa-let-7i	-1.56	0.02323
hsa-miR-210	-1.59	0.04883
miRNABrightCorner30	-1.63	0.03433
hsa-miR-29b	-1.68	0.04790
hsa-miR-18a	-2.11	0.04433
hsa-miR-7	-2.50	0.00383

Supplementary Table S5A: Ingenuity pathway analysis of differentially expressed transcripts in HUVEC cells (networks). In capitol letters genes from the imput list.

See Supplementary File 5

Supplementary Table S5B: Ingenuity pathway analysis of differentially expressed transcripts in SKMel-28 cells (networks). In capitol letters genes from the imput list.

See Supplementary File 6

Supplementary Table S6: Differentially expressed mRNAs targeted by differentially expressed miRNAs (high prediction/experimental validation) in HUVEC cells.

See Supplementary File 7
