# Supplementary material for: PDGFR-alpha inhibits melanoma growth via CXCL10/IP-10: a multi-omics approach
Source: Oncotarget. 2016 Oct 13;7(47):77257–75. doi: 10.18632/oncotarget.12629 (PMC5363585; doi:10.18632/oncotarget.12629)
Supplement: Supplementary file 2 [file oncotarget-07-77257-s002.docx]

**Supplementary Table S1. Differentially expressed transcripts in HUVEC cells overexpressing PDGFR-alpha *vs* Ad.null ctrl cells (216 genes). In bold the transcripts reported in Figure 2A.**

|  | **Target ID** | **Symbol** | **Fold-change** | **PDGFRalpha**  **AVG_Signal** | **CTRL**  **AVG_Signal** | **Diff Score** | **Entrez Gene ID** |
| --- | --- | --- | --- | --- | --- | --- | --- |
| **1** | **CCL8** | CCL8 | 13.10 | 2473.865 | 188.817 | 348.3854 | 6355 |
| **2** | **CXCL10** | CXCL10 | 9.02 | 2429.104 | 269.1889 | 348.3854 | 3627 |
| **3** | **HSPA6** | HSPA6 | 7.14 | 1269.284 | 177.6979 | 348.3854 | 3310 |
| **4** | **SNORA71C** | SNORA71C | 3.87 | 1322.24 | 341.4005 | 348.3854 | 677839 |
| **5** | **HSPA1A** | HSPA1A | 3.86 | 4242.994 | 1099.516 | 348.3854 | 3303 |
| **6** | **HSPA1B** | HSPA1B | 3.60 | 5026.834 | 1397.544 | 348.3854 | 3304 |
| **7** | **IL6** | IL6 | 3.33 | 2087.558 | 626.2772 | 348.3854 | 3569 |
| **8** | **RASD1** | RASD1 | 3.31 | 1948.983 | 589.0589 | 348.3854 | 51655 |
| **9** | **IFIT2** | IFIT2 | 2.97 | 1231.004 | 414.5984 | 348.3854 | 3433 |
| **10** | **CH25H** | CH25H | 2.96 | 2377.106 | 803.0743 | 348.3854 | 9023 |
| **11** | **ZFP36** | ZFP36 | 2.91 | 1360.285 | 468.09 | 348.3854 | 7538 |
| **12** | **STC1** | STC1 | 2.78 | 1645.947 | 591.6642 | 348.3854 | 6781 |
| **13** | **ADAMTS9** | ADAMTS9 | 2.78 | 1429.123 | 514.8175 | 348.3854 | 56999 |
| **14** | **HSPA7** | HSPA7 | 2.77 | 600.2469 | 216.5211 | 348.3854 | 3311 |
| **15** | **RHOU** | RHOU | 2.74 | 667.2073 | 243.4033 | 348.3854 | 58480 |
| **16** | **CEBPB** | CEBPB | 2.66 | 3796.689 | 1429.029 | 348.3854 | 1051 |
| **17** | **KIAA1199** | KIAA1199 | 2.65 | 320.0819 | 120.6349 | 115.1488 | 57214 |
| **18** | **GBP4** | GBP4 | 2.65 | 753.4937 | 284.7882 | 348.3854 | 115361 |
| **19** | **DARC** | DARC | 2.62 | 403.5997 | 154.1053 | 348.3854 | 2532 |
| **20** | **CEBPD** | CEBPD | 2.61 | 2426.61 | 928.933 | 348.3854 | 1052 |
| **21** | **HERC5** | HERC5 | 2.58 | 1624.346 | 629.7032 | 348.3854 | 51191 |
| **22** | **PTGS2** | PTGS2 | 2.56 | 3631.949 | 1419.328 | 348.3854 | 5743 |
| **23** | **ADAMTS1** | ADAMTS1 | 2.55 | 4878.425 | 1911.515 | 348.3854 | 9510 |
| **24** | **HS.519225** | HS.519225 | 2.42 | 533.0132 | 220.0919 | 348.3854 |  |
| **25** | **LOC653421** | LOC653421 | 2.41 | 594.428 | 246.4097 | 348.3854 | 653421 |
| **26** | **NAMPT** | NAMPT | 2.41 | 1850.183 | 768.2281 | 348.3854 | 10135 |
| **27** | **ANGPTL4** | ANGPTL4 | 2.34 | 1311.833 | 559.5942 | 149.6892 | 51129 |
| **28** | **RGS2** | RGS2 | 2.32 | 1543.594 | 664.1706 | 348.3854 | 5997 |
| **29** | **CXCL1** | CXCL1 | 2.32 | 1059.481 | 456.0234 | 69.62404 | 2919 |
| **30** | **CITED2** | CITED2 | 2.31 | 2086.681 | 902.5869 | 348.3854 | 10370 |
| **31** | **LOC387763** | LOC387763 | 2.24 | 1063.491 | 474.892 | 116.1285 | 387763 |
| **32** | **ELMOD1** | ELMOD1 | 2.23 | 687.2809 | 308.072 | 348.3854 | 55531 |
| **33** | **SHISA2** | SHISA2 | 2.22 | 912.1677 | 410.1499 | 348.3854 | 387914 |
| **34** | **CSRP2** | CSRP2 | 2.22 | 1310.082 | 589.7521 | 348.3854 | 1466 |
| **35** | **HS.156773** | HS.156773 | 2.21 | 602.337 | 272.716 | 348.3854 |  |
| **36** | **C12orf48** | C12ORF48 | 2.19 | 943.318 | 431.1773 | 173.3953 | 55010 |
| **37** | **TRIB1** | TRIB1 | 2.17 | 1399.119 | 645.5054 | 348.3854 | 10221 |
| **38** | **HTR2B** | HTR2B | 2.15 | 1068.804 | 496.6772 | 348.3854 | 3357 |
| **39** | **SELE** | SELE | 2.14 | 1134.837 | 531.3796 | 174.1927 | 6401 |
| **40** | **LOC285016** | LOC285016 | 2.12 | 230.6558 | 109.0089 | 44.62271 | 285016 |
| **41** | **CXCL2** | CXCL2 | 2.09 | 926.993 | 444.1934 | 348.3854 | 2920 |
|  | PPAP2B | PPAP2B | 2.08 | 2062.897 | 992.3543 | 232.257 | 8613 |
|  | SPOCD1 | SPOCD1 | 2.04 | 1420.276 | 696.2104 | 348.3854 | 90853 |
|  | HMOX1 | HMOX1 | 2.02 | 13937.11 | 6904.198 | 348.3854 | 3162 |
|  | NLF2 | NLF2 | 1.98 | 1261.253 | 636.5762 | 176.3592 | 388125 |
|  | HS.544325 | HS.544325 | 1.98 | 322.3344 | 163.1825 | 57.82957 |  |
|  | DNAJB1 | DNAJB1 | 1.96 | 1626.229 | 828.6574 | 348.3854 | 3337 |
|  | TMEM158 | TMEM158 | 1.93 | 716.8174 | 371.8876 | 348.3854 | 25907 |
|  | STX11 | STX11 | 1.93 | 808.7853 | 419.7914 | 348.3854 | 8676 |
|  | BATF3 | BATF3 | 1.92 | 240.5744 | 125.0984 | 38.88113 | 55509 |
|  | TNFRSF6B | TNFRSF6B | 1.90 | 788.6365 | 415.0474 | 243.9511 | 8771 |
|  | PLAC9 | PLAC9 | 1.90 | 771.5018 | 406.8659 | 133.13 | 219348 |
|  | C10orf116 | C10ORF116 | 1.88 | 1472.903 | 781.4071 | 348.3854 | 10974 |
|  | CHRNB3 | CHRNB3 | 1.88 | 318.9103 | 169.4625 | 31.32449 | 1142 |
|  | PLAUR | PLAUR | 1.87 | 1066.083 | 569.7509 | 276.6336 | 5329 |
|  | C2CD4B | C2CD4B | 1.85 | 1944.58 | 1053.591 | 348.3854 | 388125 |
|  | IFIT3 | IFIT3 | 1.84 | 516.0142 | 279.7857 | 145.8081 | 3437 |
|  | SOCS3 | SOCS3 | 1.84 | 583.3079 | 316.6783 | 182.2965 | 9021 |
|  | GADD45G | GADD45G | 1.83 | 348.8574 | 190.1293 | 41.00085 | 10912 |
|  | SPP1 | SPP1 | 1.82 | 325.2224 | 178.7762 | 61.72124 | 6696 |
|  | NRCAM | NRCAM | 1.80 | 2221.843 | 1237.222 | 348.3854 | 4897 |
|  | RARRES3 | RARRES3 | 1.79 | 816.5827 | 455.4434 | 348.3854 | 5920 |
|  | NFKBIZ | NFKBIZ | 1.79 | 549.4022 | 306.5471 | 174.1927 | 64332 |
|  | TTTY14 | TTTY14 | 1.78 | 285.7785 | 160.6476 | 43.62537 | 83869 |
|  | SOCS1 | SOCS1 | 1.78 | 296.1458 | 166.8242 | 31.74481 | 8651 |
|  | SERPINB1 | SERPINB1 | 1.77 | 1810.563 | 1022.625 | 103.5408 | 1992 |
|  | TGFB3 | TGFB3 | 1.76 | 626.8698 | 356.1242 | 120.559 | 7043 |
|  | IRF1 | IRF1 | 1.75 | 1259.088 | 720.4647 | 99.42121 | 3659 |
|  | HS.542428 | HS.542428 | 1.75 | 1496.727 | 857.3271 | 107.0185 |  |
|  | LPXN | LPXN | 1.71 | 1321.927 | 771.0556 | 348.3854 | 9404 |
|  | BCL6 | BCL6 | 1.71 | 686.1539 | 400.4472 | 174.1927 | 604 |
|  | GSDMC | GSDMC | 1.71 | 469.8209 | 275.0554 | 44.15636 | 56169 |
|  | SASS6 | SASS6 | 1.69 | 354.8333 | 209.6184 | 31.28627 | 163786 |
|  | HS.188979 | HS.188979 | 1.69 | 1115.427 | 659.8743 | 348.3854 |  |
|  | IL1RL1 | IL1RL1 | 1.69 | 681.8767 | 404.3763 | 174.1927 | 9173 |
|  | AADACL1 | AADACL1 | 1.68 | 1017.307 | 604.1798 | 202.1422 | 57552 |
|  | RPL29 | RPL29 | 1.68 | 2677.768 | 1593.07 | 348.3854 | 6159 |
|  | BMP2 | BMP2 | 1.66 | 632.7479 | 380.48 | 121.4916 | 650 |
|  | SELL | SELL | 1.66 | 536.0432 | 322.8894 | 97.3898 | 6402 |
|  | MALL | MALL | 1.65 | 8714.662 | 5265.829 | 67.27371 | 7851 |
|  | SLC25A28 | SLC25A28 | 1.65 | 3031.725 | 1841.599 | 348.3854 | 81894 |
|  | LOC440359 | LOC440359 | 1.64 | 8324.935 | 5081.313 | 115.1914 | 440359 |
|  | SNCG | SNCG | 1.64 | 692.149 | 422.972 | 126.3559 | 6623 |
|  | STC2 | STC2 | 1.63 | 1044.565 | 639.025 | 106.8904 | 8614 |
|  | EPSTI1 | EPSTI1 | 1.62 | 1103.658 | 679.4168 | 348.3854 | 94240 |
|  | CDH11 | CDH11 | 1.62 | 801.8773 | 493.6681 | 69.77036 | 1009 |
|  | GABBR2 | GABBR2 | 1.62 | 3709.588 | 2296.258 | 348.3854 | 9568 |
|  | HIST1H2BK | HIST1H2BK | 1.61 | 4882.563 | 3025.113 | 348.3854 | 85236 |
|  | KRT19 | KRT19 | 1.61 | 1678.17 | 1044.739 | 348.3854 | 3880 |
|  | RNF217 | RNF217 | 1.60 | 581.5289 | 363.2397 | 94.97675 | 154214 |
|  | NFIL3 | NFIL3 | 1.60 | 730.5414 | 456.6247 | 70.91409 | 4783 |
|  | TBC1D2 | TBC1D2 | 1.60 | 564.6345 | 353.0592 | 90.80476 | 55357 |
|  | LOC100144604 | LOC100144604 | 1.59 | 328.011 | 205.8365 | 34.57614 | 100144604 |
|  | FAM107A | FAM107A | 1.59 | 2452.116 | 1540.823 | 348.3854 | 11170 |
|  | MYC | MYC | 1.59 | 2449.507 | 1539.504 | 208.7442 | 4609 |
|  | HES2 | HES2 | 1.59 | 411.5778 | 259.2205 | 40.74406 | 54626 |
|  | ACTG2 | ACTG2 | 1.58 | 877.5808 | 553.7744 | 348.3854 | 72 |
|  | NCOA7 | NCOA7 | 1.58 | 3593.123 | 2277.304 | 348.3854 | 135112 |
|  | GBP1 | GBP1 | 1.58 | 2109.93 | 1337.653 | 193.2685 | 2633 |
|  | FAM46A | FAM46A | 1.58 | 539.4276 | 342.3497 | 80.64607 | 55603 |
|  | TAP1 | TAP1 | 1.57 | 3839.525 | 2444.2 | 133.13 | 6890 |
|  | NAP1L5 | NAP1L5 | 1.56 | 1302.086 | 836.9548 | 348.3854 | 266812 |
|  | PTGER4 | PTGER4 | 1.56 | 1068.866 | 687.3373 | 348.3854 | 5734 |
|  | SLC7A11 | SLC7A11 | 1.55 | 976.2284 | 628.2138 | 133.13 | 23657 |
|  | CXCR7 | CXCR7 | 1.55 | 1236.278 | 797.9034 | 199.3699 | 57007 |
|  | ERRFI1 | ERRFI1 | 1.55 | 1583.623 | 1024.608 | 348.3854 | 54206 |
|  | GPR37 | GPR37 | 1.54 | 439.2119 | 285.861 | 37.60378 | 2861 |
|  | SEMA3F | SEMA3F | 1.53 | 1737.886 | 1134.691 | 348.3854 | 6405 |
|  | SPRED1 | SPRED1 | 1.53 | 1403.703 | 918.0828 | 110.8696 | 161742 |
|  | IL8 (CXCL8) | CXCL8 | 1.53 | 939.0421 | 614.22 | 85.24998 | 3576 |
|  | HS.543887 | HS.543887 | 1.53 | 713.3662 | 466.8415 | 54.63805 |  |
|  | PMAIP1 | PMAIP1 | 1.53 | 673.7888 | 441.1777 | 67.4978 | 5366 |
|  | MMP1 | MMP1 | 1.53 | 7343.754 | 4810.524 | 108.3904 | 4312 |
|  | DOCK10 | DOCK10 | 1.51 | 930.3985 | 616.7472 | 122.3566 | 55619 |
|  | HSPA4L | HSPA4L | 1.51 | 537.5208 | 356.4062 | 46.75764 | 22824 |
|  | GPC1 | GPC1 | 1.51 | 664.005 | 440.8 | 86.49526 | 2817 |
|  | TMEM163 | TMEM163 | 1.51 | 430.1455 | 285.7786 | 32.72919 | 81615 |
|  | PTPN13 | PTPN13 | 1.50 | 381.8042 | 253.8625 | 38.57478 | 5783 |
|  | SLC4A7 | SLC4A7 | 1.50 | 947.8717 | 630.7748 | 200.0603 | 9497 |
|  | CYTL1 | CYTL1 | 1.50 | 1067.222 | 712.0426 | 53.81984 | 54360 |
|  | ANKRD1 | ANKRD1 | 1.50 | 1777.262 | 1186.973 | 348.3854 | 27063 |
|  | KCNAB1 | KCNAB1 | 1.50 | 376.9337 | 251.9636 | 87.09636 | 7881 |
|  | FCGR3B | FCGR3B | 1.50 | 427.0267 | 285.6233 | 37.34796 | 2215 |
|  | ITGA10 | ITGA10 | -1.50 | 2394.577 | 3580.369 | -74.92358 | 8515 |
|  | NCAPD2 | NCAPD2 | -1.50 | 355.6927 | 532.3262 | -61.50334 | 9918 |
|  | TMEM204 | TMEM204 | -1.50 | 1228.681 | 1839.668 | -154.3865 | 79652 |
|  | MTMR10 | MTMR10 | -1.50 | 2423.931 | 3634.115 | -107.5807 | 54893 |
|  | HSPA2 | HSPA2 | -1.50 | 358.4951 | 538.6589 | -35.42812 | 3306 |
|  | SNORD3D | SNORD3D | -1.50 | 847.1593 | 1273.886 | -94.73955 | 780854 |
|  | KHDRBS3 | KHDRBS3 | -1.51 | 665.299 | 1003.497 | -74.10656 | 10656 |
|  | C8orf4 | C8ORF4 | -1.51 | 5424.098 | 8194.839 | -251.9127 | 56892 |
|  | TM4SF18 | TM4SF18 | -1.51 | 4639.523 | 7024.123 | -160.3103 | 116441 |
|  | AQP1 | AQP1 | -1.52 | 342.5226 | 519.5361 | -57.59844 | 358 |
|  | GAS6 | GAS6 | -1.52 | 928.0825 | 1409.361 | -92.14185 | 2621 |
|  | HIST1H4C | HIST1H4C | -1.52 | 3853.117 | 5851.506 | -238.5275 | 8364 |
|  | CTSF | CTSF | -1.52 | 646.5655 | 982.7635 | -70.30206 | 8722 |
|  | GGA2 | GGA2 | -1.52 | 665.2257 | 1011.391 | -85.23268 | 23062 |
|  | CDCA5 | CDCA5 | -1.52 | 229.2901 | 349.0791 | -35.52206 | 113130 |
|  | TMEM9 | TMEM9 | -1.53 | 428.1123 | 654.059 | -92.2418 | 252839 |
|  | OLFML2A | OLFML2A | -1.53 | 230.6612 | 353.496 | -37.45888 | 169611 |
|  | SPC24 | SPC24 | -1.53 | 246.0193 | 377.1889 | -42.00698 | 147841 |
|  | MT1A | MT1A | -1.54 | 6442.519 | 9904.758 | -91.61196 | 4489 |
|  | SNCAIP | SNCAIP | -1.54 | 659.4131 | 1014.266 | -94.34621 | 9627 |
|  | MEF2C | MEF2C | -1.54 | 407.2249 | 626.3832 | -70.62501 | 4208 |
|  | LOC441019 | LOC441019 | -1.54 | 618.1904 | 951.7415 | -140.3107 | 441019 |
|  | ARL6IP1 | ARL6IP1 | -1.54 | 2357.577 | 3635.436 | -111.7457 | 23204 |
|  | NCAPG | NCAPG | -1.54 | 316.3051 | 487.774 | -44.03829 | 64151 |
|  | KIAA0430 | KIAA0430 | -1.54 | 893.8279 | 1378.385 | -105.7636 | 9665 |
|  | AURKA | AURKA | -1.55 | 317.0707 | 490.7713 | -62.28708 | 6790 |
|  | BMP4 | BMP4 | -1.55 | 1669.886 | 2586.796 | -110.9661 | 652 |
|  | STS-1 | STS-1 | -1.55 | 352.1387 | 545.5724 | -77.51876 | 84959 |
|  | AIDA | AIDA | -1.56 | 1884.74 | 2938.234 | -97.81354 | 64853 |
|  | SNHG7 | SNHG7 | -1.56 | 1350.71 | 2106.575 | -156.4526 | 84973 |
|  | CIRBP | CIRBP | -1.56 | 3886.67 | 6072.509 | -285.7834 | 1153 |
|  | TPX2 | TPX2 | -1.59 | 240.3816 | 381.7953 | -46.04352 | 22974 |
|  | CRYAB | CRYAB | -1.59 | 397.7333 | 634.2798 | -92.91683 | 1410 |
|  | EFCAB4A | EFCAB4A | -1.60 | 336.8651 | 539.1141 | -45.43741 | 283229 |
|  | PLLP | PLLP | -1.60 | 369.0893 | 591.5201 | -69.85994 | 51090 |
|  | SNORD13 | SNORD13 | -1.60 | 2026.785 | 3248.954 | -285.0349 | 692084 |
|  | SLC27A1 | SLC27A1 | -1.60 | 514.092 | 824.734 | -36.78908 | 376497 |
|  | MARCH4 | MARCH4 | -1.61 | 830.7968 | 1333.717 | -214.6133 | 57574 |
|  | SERTAD4 | SERTAD4 | -1.61 | 223.6239 | 359.4477 | -36.68963 | 56256 |
|  | LOC643733 | LOC643733 | -1.61 | 1028.23 | 1655.412 | -125.6814 | 643733 |
|  | C10orf10 | C10ORF10 | -1.61 | 4163.272 | 6708.217 | -329.4458 | 11067 |
|  | CDKN3 | CDKN3 | -1.62 | 222.5998 | 361.649 | -40.13385 | 1033 |
|  | ACSS1 | ACSS1 | -1.63 | 315.1807 | 513.857 | -52.64581 | 84532 |
|  | LOC648470 | LOC648470 | -1.63 | 2357.762 | 3845.71 | -265.9766 | 648470 |
|  | SNHG6 | SNHG6 | -1.63 | 1649.962 | 2692.598 | -257.4114 | 641638 |
|  | UACA | UACA | -1.64 | 269.6404 | 442.377 | -86.86629 | 55075 |
|  | FNBP1 | FNBP1 | -1.64 | 1094.319 | 1795.739 | -154.6297 | 23048 |
|  | KIAA1274 | KIAA1274 | -1.65 | 972.9274 | 1600.943 | -87.65352 | 27143 |
|  | NOTCH4 | NOTCH4 | -1.65 | 886.4684 | 1466.154 | -203.6712 | 4855 |
|  | MFGE8 | MFGE8 | -1.66 | 2600.915 | 4318.978 | -170.4357 | 4240 |
|  | PGF | PGF | -1.66 | 875.9857 | 1455.617 | -43.03851 | 5228 |
|  | C1orf133 | C1ORF133 | -1.67 | 505.1035 | 841.3473 | -140.018 | 574036 |
| **42** | **CCNA2** | CCNA2 | -1.72 | 276.2928 | 474.7784 | -31.35818 | 890 |
| **43** | **GNG7** | GNG7 | -1.72 | 202.2682 | 348.0623 | -52.7914 | 2788 |
| **44** | **IRF6** | IRF6 | -1.73 | 175.3983 | 302.6264 | -37.65633 | 3664 |
| **45** | **ASNA1** | ASNA1 | -1.76 | 176.6057 | 310.7101 | -39.38641 | 439 |
| **46** | **CD34** | CD34 | -1.76 | 3342.315 | 5885.742 | -140.1524 | 947 |
| **47** | **SLC30A3** | SLC30A3 | -1.76 | 890.2596 | 1568.582 | -326.828 | 7781 |
| **48** | **ANTXR1** | ANTXR1 | -1.77 | 283.9328 | 501.1447 | -95.23249 | 84168 |
| **49** | **FAM84B** | FAM84B | -1.77 | 697.704 | 1238.002 | -290.9572 | 157638 |
| **50** | **CHRNA1** | CHRNA1 | -1.78 | 208.9427 | 371.0309 | -71.51588 | 1134 |
| **51** | **NEDD4L** | NEDD4L | -1.79 | 448.6278 | 802.2427 | -153.6289 | 23327 |
| **52** | **LMCD1** | LMCD1 | -1.80 | 437.1912 | 787.8571 | -88.06458 | 29995 |
| **53** | **CXCR4** | CXCR4 | -1.81 | 185.0549 | 335.1599 | -93.44619 | 7852 |
| **54** | **ANLN** | ANLN | -1.82 | 167.1849 | 303.8148 | -36.17452 | 54443 |
| **55** | **NUSAP1** | NUSAP1 | -1.82 | 202.174 | 367.6772 | -82.98405 | 51203 |
| **56** | **RHOB** | RHOB | -1.83 | 3348.035 | 6130.726 | -345.9821 | 388 |
| **57** | **PHGDH** | PHGDH | -1.84 | 410.6364 | 755.1498 | -169.2175 | 26227 |
| **58** | **CLIP3** | CLIP3 | -1.85 | 1546.593 | 2853.992 | -345.9821 | 25999 |
| **59** | **SEMA3G** | SEMA3G | -1.86 | 123.305 | 229.6314 | -30.69116 | 56920 |
| **60** | **NTN4** | NTN4 | -1.89 | 962.3099 | 1815.139 | -189.9257 | 59277 |
| **61** | **RN5S9** | RN5S9 | -1.90 | 727.707 | 1383.554 | -138.774 | 1E+08 |
| **62** | **LOC730432** | LOC730432 | -1.96 | 780.6429 | 1528.055 | -345.9821 | 730432 |
| **63** | **PDGFB** | PDGFB | -1.97 | 791.5229 | 1555.49 | -172.701 | 5155 |
| **64** | **SH3BP4** | SH3BP4 | -2.03 | 1719.061 | 3486.625 | -345.9821 | 23677 |
| **65** | **AIF1L** | AIF1L | -2.03 | 1990.731 | 4038.52 | -340.3875 | 83543 |
| **66** | **ASPM** | ASPM | -2.03 | 203.5716 | 413.6488 | -75.14427 | 259266 |
| **67** | **FBXO32** | FBXO32 | -2.04 | 436.0196 | 891.1733 | -201.0936 | 114907 |
| **68** | **UBE2C** | UBE2C | -2.06 | 187.1234 | 386.0821 | -138.7253 | 11065 |
| **69** | **C9orf58** | C9ORF58 | -2.11 | 344.1126 | 727.5388 | -282.6154 | 83543 |
| **70** | **TK1** | TK1 | -2.12 | 184.8298 | 391.6805 | -91.14362 | 7083 |
| **71** | **DLGAP5** | DLGAP5 | -2.12 | 187.144 | 397.1775 | -103.6294 | 9787 |
| **72** | **ESM1** | ESM1 | -2.16 | 3686.612 | 7968.691 | -310.3329 | 11082 |
| **73** | **AURKB** | AURKB | -2.17 | 134.0037 | 290.555 | -71.61134 | 9212 |
| **74** | **KIF20A** | KIF20A | -2.18 | 208.9699 | 455.5463 | -159.6739 | 10112 |
| **75** | **MYCN** | MYCN | -2.19 | 268.2288 | 586.2256 | -179.2081 | 4613 |
| **76** | **TOP2A** | TOP2A | -2.43 | 193.5946 | 470.5361 | -93.73664 | 7153 |
| **77** | **CCNB2** | CCNB2 | -2.47 | 213.604 | 527.5364 | -220.0954 | 9133 |
| **78** | **CEP55** | CEP55 | -2.54 | 184.7279 | 470.0138 | -209.6496 | 55165 |
| **79** | **PRC1** | PRC1 | -2.57 | 321.3301 | 824.7139 | -345.9821 | 9055 |
| **80** | **CDC20** | CDC20 | -2.67 | 318.6231 | 851.5031 | -345.9821 | 991 |
| **81** | **FABP4** | FABP4 | -2.90 | 1825.082 | 5299.119 | -345.9821 | 2167 |
| **82** | **GJA4** | GJA4 | -3.71 | 488.2987 | 1812.886 | -345.9821 | 2701 |
