# Supplementary material for: PDGFR-alpha inhibits melanoma growth via CXCL10/IP-10: a multi-omics approach
Source: Oncotarget. 2016 Oct 13;7(47):77257–75. doi: 10.18632/oncotarget.12629 (PMC5363585; doi:10.18632/oncotarget.12629)
Supplement: Supplementary file 3 [file oncotarget-07-77257-s003.docx]

**Supplementary Table S2. Differentially expressed transcripts in SKMel-28 cells overexpressing PDGFR alpha vs Ad.null ctrl cells (107 genes). In bold the transcripts reported in Figure 2B.**

|  | Target ID | Symbol | Fold-change | PDGFRalpha  AVG_Signal | CTRL  AVG_Signal | Diff Score | Entrez Gene ID |
| --- | --- | --- | --- | --- | --- | --- | --- |
| **1** | **CXCL10** | CXCL10 | 23.43 | 3308.043 | 141.2144 | 346.6245 | 3627 |
| **2** | **IFIT2** | IFIT2 | 23.36 | 4660.022 | 199.4578 | 346.6245 | 3433 |
| **3** | **GBP4** | GBP4 | 10.74 | 1832.032 | 170.5902 | 346.6245 | 115361 |
| **4** | **MX1** | MX1 | 9.99 | 4390.693 | 439.4833 | 346.6245 | 4599 |
| **5** | **IFI44L** | IFI44L | 8.79 | 970.9132 | 110.4605 | 346.6245 | 10964 |
| **6** | **IFIT3** | IFIT3 | 7.34 | 1468.561 | 200.075 | 272.0003 | 3437 |
| **7** | **INDO** | INDO | 6.54 | 656.3779 | 100.4081 | 346.6245 | 3620 |
| **8** | **EPSTI1** | EPSTI1 | 5.91 | 859.738 | 145.3607 | 346.6245 | 94240 |
| **9** | **IDO1** | IDO1 | 5.85 | 866.2747 | 148.07 | 346.6245 | 3620 |
| **10** | **IRF1** | IRF1 | 5.57 | 1293.802 | 232.3902 | 346.6245 | 3659 |
| **11** | **IFIT1** | IFIT1 | 5.10 | 889.1 | 174.4919 | 175.3439 | 3434 |
| **12** | **RSAD2** | RSAD2 | 4.78 | 720.7943 | 150.639 | 346.6245 | 91543 |
| **13** | **ITK** | ITK | 4.22 | 684.424 | 162.0729 | 346.6245 | 3702 |
| **14** | **IFIH1** | IFIH1 | 3.76 | 758.9341 | 201.9699 | 346.6245 | 64135 |
| **15** | **GBP1** | GBP1 | 3.71 | 2287.292 | 616.1121 | 346.6245 | 2633 |
| **16** | **SNORA71C** | SNORA71C | 3.29 | 1778.731 | 541.0341 | 346.6245 | 677839 |
| **17** | **PRIC285** | PRIC285 | 3.16 | 1904.729 | 602.2907 | 346.6245 | 85441 |
| **18** | **CXCL9** | CXCL9 | 3.11 | 322.1615 | 103.7008 | 116.1402 | 4283 |
| **19** | **SAMD9L** | SAMD9L | 2.97 | 473.4832 | 159.3343 | 346.6245 | 219285 |
| **20** | **IFITM1** | IFITM1 | 2.97 | 493.8241 | 166.3165 | 346.6245 | 8519 |
| **21** | **SAMD9** | SAMD9 | 2.89 | 946.1028 | 327.6563 | 346.6245 | 54809 |
| **22** | **ISG15** | ISG15 | 2.87 | 1217.071 | 424.7867 | 346.6245 | 9636 |
| **23** | **MX2** | MX2 | 2.84 | 452.2535 | 159.4909 | 346.6245 | 4600 |
| **24** | **TNFSF10** | TNFSF10 | 2.77 | 274.0374 | 98.83588 | 50.96371 | 8743 |
| **25** | **RARRES3** | RARRES3 | 2.72 | 566.3142 | 208.4079 | 346.6245 | 5920 |
| **26** | **LOC653421** | LOC653421 | 2.71 | 793.2027 | 292.5007 | 346.6245 | 653421 |
| **27** | **TAP1** | TAP1 | 2.61 | 2144.837 | 820.4358 | 346.6245 | 6890 |
| **28** | **NEXN** | NEXN | 2.58 | 534.9036 | 207.654 | 346.6245 | 91624 |
| **29** | **HS.156773** | HS.156773 | 2.56 | 784.1197 | 306.6885 | 346.6245 |  |
| **30** | **INO80B** | INO80B | 2.52 | 982.3674 | 389.2159 | 346.6245 | 83444 |
| **31** | **HS.519225** | HS.519225 | 2.48 | 696.8437 | 280.6672 | 346.6245 |  |
| **32** | **IFITM3** | IFITM3 | 2.44 | 2342.63 | 958.6329 | 346.6245 | 10410 |
| **33** | **CHRNB3** | CHRNB3 | 2.44 | 416.9509 | 170.8619 | 114.4407 | 1142 |
| **34** | **IFITM2** | IFITM2 | 2.42 | 2028.568 | 838.1506 | 346.6245 | 10581 |
| **35** | **OAS1** | OAS1 | 2.40 | 703.0534 | 292.6595 | 203.2907 | 4938 |
| **36** | **IFI27** | IFI27 | 2.37 | 342.8763 | 144.5065 | 62.05595 | 3429 |
| **37** | **EGR2** | EGR2 | 2.33 | 970.1038 | 416.7461 | 346.6245 | 1959 |
| **38** | **NPTX2** | NPTX2 | 2.31 | 663.6859 | 287.1572 | 346.6245 | 4885 |
| **39** | **USP18** | USP18 | 2.30 | 634.5774 | 276.468 | 346.6245 | 11274 |
| **40** | **TTTY14** | TTTY14 | 2.29 | 369.6456 | 161.7253 | 31.01094 | 83869 |
| **41** | **GBP2** | GBP2 | 2.28 | 1225.131 | 536.5606 | 346.6245 | 2634 |
| **42** | **LOC100128274** | LOC100128274 | 2.24 | 440.3523 | 196.4476 | 346.6245 | 1E+08 |
| **43** | **IFI44** | IFI44 | 2.21 | 1406.606 | 636.5282 | 346.6245 | 10561 |
|  | XIRP1 | XIRP1 | 2.19 | 213.4196 | 97.52424 | 33.67608 | 165904 |
|  | OASL | OASL | 2.17 | 291.4395 | 134.5152 | 173.3124 | 8638 |
|  | HMOX1 | HMOX1 | 2.15 | 13072.96 | 6089.139 | 53.76565 | 3162 |
|  | HSPA1B | HSPA1B | 2.14 | 9571.571 | 4474.115 | 346.6245 | 3304 |
|  | SOCS3 | SOCS3 | 2.06 | 397.35 | 192.9631 | 173.3124 | 9021 |
|  | OAS2 | OAS2 | 2.00 | 482.6433 | 240.9699 | 97.09855 | 4939 |
|  | ACTG2 | ACTG2 | 1.99 | 4224.625 | 2118.382 | 346.6245 | 72 |
|  | PARP14 | PARP14 | 1.93 | 1421.067 | 736.9875 | 346.6245 | 54625 |
|  | HSPA6 | HSPA6 | 1.91 | 499.2726 | 261.8994 | 179.8034 | 3310 |
|  | CCL3L3 | CCL3L3 | 1.89 | 2177.779 | 1149.795 | 346.6245 | 414062 |
|  | CCL3 | CCL3 | 1.89 | 2069.765 | 1094.393 | 346.6245 | 6348 |
|  | SOCS1 | SOCS1 | 1.86 | 284.435 | 152.8676 | 43.62544 | 8651 |
|  | LOC400759 | LOC400759 | 1.85 | 322.2134 | 173.8521 | 45.72334 | 400759 |
|  | RIMS4 | RIMS4 | 1.84 | 399.5766 | 217.5379 | 62.82683 | 140730 |
|  | HERC5 | HERC5 | 1.83 | 3435.335 | 1881.545 | 346.6245 | 51191 |
|  | RASGRP3 | RASGRP3 | 1.79 | 306.4797 | 170.9614 | 45.27308 | 25780 |
|  | ACTA2 | ACTA2 | 1.79 | 284.7877 | 158.8717 | 39.07644 | 59 |
|  | TXNIP | TXNIP | 1.78 | 623.4149 | 349.5865 | 71.82525 | 10628 |
|  | TNFSF13B | TNFSF13B | 1.75 | 782.4677 | 445.8791 | 101.683 | 10673 |
|  | ASPHD2 | ASPHD2 | 1.74 | 565.9939 | 325.3607 | 60.10406 | 57168 |
|  | PMAIP1 | PMAIP1 | 1.73 | 573.761 | 331.2181 | 175.7091 | 5366 |
|  | LOC100008588 | LOC100008588 | 1.72 | 14028.38 | 8177.004 | 346.6245 | 1E+08 |
|  | CCL3L1 | CCL3L1 | 1.71 | 729.9374 | 426.5772 | 231.0831 | 6349 |
|  | XAF1 | XAF1 | 1.71 | 365.1407 | 213.4781 | 174.4547 | 54739 |
|  | MLKL | MLKL | 1.70 | 373.6216 | 219.7655 | 89.326 | 197259 |
|  | ZFP36 | ZFP36 | 1.69 | 1058.65 | 627.5652 | 346.6245 | 7538 |
|  | NCOA7 | NCOA7 | 1.68 | 1762.896 | 1046.581 | 119.9137 | 135112 |
|  | IRF9 | IRF9 | 1.68 | 1320.732 | 787.6886 | 346.6245 | 10379 |
|  | HSPA1A | HSPA1A | 1.68 | 7265.217 | 4333.384 | 37.51991 | 3303 |
|  | SP110 | SP110 | 1.67 | 1027.531 | 614.8955 | 121.0562 | 3431 |
|  | DACT3 | DACT3 | 1.67 | 401.2542 | 240.4815 | 58.95867 | 147906 |
|  | ZNF432 | ZNF432 | 1.66 | 480.1341 | 289.6382 | 65.54475 | 9668 |
|  | LAP3 | LAP3 | 1.66 | 2605.022 | 1571.981 | 124.8368 | 51056 |
|  | MAFB | MAFB | 1.65 | 343.7902 | 207.8747 | 42.9789 | 9935 |
|  | SASS6 | SASS6 | 1.65 | 491.0318 | 298.1101 | 46.29673 | 163786 |
|  | BASP1 | BASP1 | 1.61 | 372.3744 | 230.9971 | 45.09619 | 10409 |
|  | PARP12 | PARP12 | 1.59 | 655.8647 | 412.7074 | 105.3379 | 64761 |
|  | NT5C3 | NT5C3 | 1.58 | 2423.715 | 1533.701 | 210.143 | 51251 |
|  | HS.567036 | HS.567036 | 1.57 | 350.2433 | 223.2394 | 35.86374 |  |
|  | PLSCR4 | PLSCR4 | 1.57 | 1476.85 | 941.4528 | 346.6245 | 57088 |
|  | MAF | MAF | 1.56 | 924.2567 | 591.7285 | 173.3124 | 4094 |
|  | ICAM1 | ICAM1 | 1.56 | 1219.164 | 783.002 | 58.4301 | 3383 |
|  | SERPINH1 | SERPINH1 | 1.55 | 989.175 | 636.9448 | 39.92863 | 871 |
|  | SNORD3A | SNORD3A | 1.55 | 625.2915 | 403.3791 | 66.76362 | 780851 |
|  | GABARAPL1 | GABARAPL1 | 1.55 | 822.8862 | 531.2515 | 84.02749 | 23710 |
|  | HS.188979 | HS.188979 | 1.55 | 1051.973 | 680.1433 | 117.6927 |  |
|  | IFI35 | IFI35 | 1.54 | 1748.904 | 1132.717 | 346.6245 | 3430 |
|  | SNORD3D | SNORD3D | 1.54 | 1096.885 | 710.6122 | 71.8425 | 780854 |
|  | HIST1H2BK | HIST1H2BK | 1.54 | 2697.288 | 1751.592 | 53.69638 | 85236 |
|  | LOC644254 | LOC644254 | 1.54 | 455.0346 | 295.9531 | 44.80044 | 644254 |
|  | FST | FST | 1.53 | 340.8603 | 223.0343 | 55.34366 | 10468 |
|  | PI15 | PI15 | 1.51 | 1686.664 | 1119.049 | 86.5814 | 51050 |
|  | CEBPD | CEBPD | 1.51 | 653.59 | 433.9598 | 47.3815 | 1052 |
|  | GCLM | GCLM | 1.50 | 1780.24 | 1184.58 | 178.6022 | 2730 |
|  | TMEM173 | TMEM173 | 1.50 | 736.3071 | 491.6949 | 93.0485 | 340061 |
| **44** | **FLJ10374** | FLJ10374 | -1.55 | 338.9999 | 524.1264 | -48.51517 | 55702 |
| **45** | **SPRR2F** | SPRR2F | -1.62 | 261.1242 | 423.7527 | -44.93422 | 6705 |
| **46** | **HIST1H4C** | HIST1H4C | -1.65 | 9200.929 | 15205.09 | -215.4042 | 8364 |
| **47** | **C6orf125** | C6ORF125 | -1.69 | 1225.771 | 2073.413 | -167.2793 | 84300 |
| **48** | **LYPLA2** | LYPLA2 | -1.75 | 441.2485 | 774.3582 | -39.84994 | 11313 |
| **49** | **SPRR2D** | SPRR2D | -1.90 | 462.8776 | 881.6874 | -171.8608 | 6703 |
| **50** | **RDH5** | RDH5 | -2.07 | 349.612 | 722.597 | -192.5684 | 5959 |
| **51** | **SNHG6** | SNHG6 | -2.29 | 2364.964 | 5423.54 | -249.6656 | 641638 |
| **52** | **IL1B** | IL1B | -4.78 | 133.8117 | 639.4447 | -343.7215 | 3553 |
