# Supplementary material for: PDGFR-alpha inhibits melanoma growth via CXCL10/IP-10: a multi-omics approach
Source: Oncotarget. 2016 Oct 13;7(47):77257–75. doi: 10.18632/oncotarget.12629 (PMC5363585; doi:10.18632/oncotarget.12629)
Supplement: Supplementary file 4 [file oncotarget-07-77257-s004.docx]

**Supplementary Table S3. Differentially expressed miRNAs in HUVEC cells overexpressing PDGFR-alpha *vs* Ad.null ctrl cells.**

| **miRNA** | **Fold-change** | ***p-value*** |
| --- | --- | --- |
| hsa-miR-630 | 12.82 | *0.00059* |
| hsa-miR-575 | 5.86 | *0.00001* |
| hsa-miR-1290 | 4.26 | *0.00053* |
| hsa-miR-125a-3p | 4.04 | *0.00006* |
| hsa-miR-1207-5p | 3.45 | *0.00135* |
| hsa-miR-1246 | 3.20 | *0.00151* |
| hsa-miR-718 | 2.97 | *0.04040* |
| hsa-miR-1225-5p | 2.69 | *0.00434* |
| hsa-miR-1268 | 2.66 | *0.00491* |
| hsa-miR-134 | 2.45 | *0.00019* |
| hsa-miR-638 | 2.43 | *0.00004* |
| hsa-miR-1202 | 2.42 | *0.02702* |
| hsa-miR-572 | 1.70 | *0.02266* |
| hsa-miR-320c | 1.53 | *0.00871* |
| hsa-miR-320d | -1.52 | *0.00933* |
| hsa-miR-424 | -1.53 | *0.02392* |
| hsa-miR-30a | -1.54 | *0.03438* |
| hsa-miR-151-3p | -1.57 | *0.04599* |
| hsa-miR-25 | -1.57 | *0.03679* |
| hsa-miR-27b | -1.58 | *0.04400* |
| hsa-miR-17 | -1.63 | *0.04107* |
| hsa-miR-106b | -1.67 | *0.03400* |
| hsa-miR-214 | -1.70 | *0.03848* |
| hsa-miR-130b | -1.71 | *0.03864* |
| hsa-miR-29a | -1.71 | *0.02498* |
| hsa-miR-331-3p | -1.74 | *0.02754* |
| hsa-miR-574-5p | -1.77 | *0.04146* |
| hsa-miR-26a | -1.77 | *0.01416* |
| hsa-miR-27a | -1.78 | *0.04049* |
| hsa-miR-34a | -1.81 | *0.03256* |
| hsa-miR-125a-5p | -1.84 | *0.04927* |
| hsa-miR-181b | -1.86 | *0.03700* |
| hsa-miR-361-5p | -1.88 | *0.04326* |
| hsa-miR-301b | -1.91 | *0.00024* |
| hsa-miR-107 | -1.92 | *0.03990* |
| hsa-miR-221 | -1.93 | *0.01615* |
| hsa-miR-376c | -1.96 | *0.04010* |
| hsa-miR-29b | -1.96 | *0.00972* |
| hsa-let-7b | -1.99 | *0.03413* |
| hsa-miR-130a | -2.00 | *0.01308* |
| hsa-miR-140-3p | -2.04 | *0.04516* |
| hsa-miR-103 | -2.05 | *0.02132* |
| hsa-miR-99b | -2.10 | *0.04386* |
| hsa-miR-93 | -2.11 | *0.01913* |
| hsa-miR-24 | -2.11 | *0.00577* |
| hsa-miR-216a | -2.20 | *0.01119* |
| hsa-miR-494 | -2.47 | *0.01853* |
| hsa-miR-379 | -2.57 | *0.00666* |
| hsa-miR-217 | -2.60 | *0.01264* |
| hsa-miR-1973 | -2.91 | *0.02315* |
| hsa-miR-185 | -2.96 | *0.04415* |
| hsa-miR-342-3p | -3.31 | *0.00437* |
| hsa-miR-324-5p | -3.94 | *0.03989* |
| hsa-miR-503 | -6.39 | *0.01611* |
