# Supplementary material for: PDGFR-alpha inhibits melanoma growth via CXCL10/IP-10: a multi-omics approach
Source: Oncotarget. 2016 Oct 13;7(47):77257–75. doi: 10.18632/oncotarget.12629 (PMC5363585; doi:10.18632/oncotarget.12629)
Supplement: Supplementary file 5 [file oncotarget-07-77257-s005.docx]

**Supplementary Table S5A. Ingenuity Pathway Analysis of differentially expressed transcripts in HUVEC cells (networks). In capitol letters genes from the imput list.**

| **ID** | **Molecules in Network** | **Score** | **Focus Molecules** | **Top Diseases and Functions** |
| --- | --- | --- | --- | --- |
| 1 | ACKR1,ACKR3,Ap1,APC (complex),AURKA,AURKB,BMP2,BMP4,Cbp/p300,CDC20,CEBPD,Creb,CRYAB,Ctbp,CXCR4,Cyclin E,DLGAP5,E2f,ERK1/2,IL1RL1,MMP1,MYCN,NCAPG,NUSAP1,PGF,PLAUR,PTGER4,Ras homolog,Rock,SAA,SNCG,SPP1,TMSB4,TPX2,UBE2C | 35 | 23 | Cancer, Organismal Injury and Abnormalities, Tumor Morphology |
| 2 | ACTG2,ADAMTS1,Alpha catenin, ANGPTL4, BCL6, CD3,CDH11,CH25H,CITED2,DNAJB1,FSH,GBP1,GPR37,Growth hormone,Gsk3,HERC5,HIST1H4C,Histone h3,Histone h4, Hsp70, HSPA1A/HSPA1B, IgG,Jnk,Lh,MYC,NFIL3,PMAIP1,Rac,RNA polymerase II,SH3BP4,TGFB3,TK1,TM4SF18,TOP2A,TRIB1 | 33 | 22 | Neurological Disease, Cell Death and Survival, Cancer |
| 3 | BCR (complex), CCL8, CCNB2, Cdk, CXCL2, CXCL10, FCGR3A/FCGR3B,IFIT2,IFIT3,IFN Beta,Ifn gamma,Iga,IL1,IL12 (complex), Immunoglobulin, Interferon alpha, IRF1, LDL,NAMPT,NFkB (complex),NFKBIZ,Oas,PDGF BB,PI3K (complex), PLPP3, RARRES3, RGS2, RHOB,SELL,SOCS1,SOCS3,TAP1,TCR,Tlr,ZFP36 | 27 | 19 | Antimicrobial Response, Inflammatory Response, Immunological Disease |
| 4 | ACKR3,AGTR1,ALOX5AP,BST2,CDKN1A,CEMIP,CSRP2,DNM1,ELMOD1,EZH2,FNBP1,HSF1,HSF2,HSPA6,HSPA7,HSPA1A/HSPA1B,HSPA4L,HTR2B,IFI16,KCNAB1,LGALS3BP,MAPK1,MAPK13,MECP2,MEF2C,MT1A,NCOA7,NR3C1,OLFML2A,RARRES3,TMEM158,TP53I3,TRAF3,WDR26,ZC3HAV1 | 23 | 17 | Hair and Skin Development and Function, Organ Morphology, Tissue Morphology |
| 5 | ADIRF,AIF1L,Akt,ANKRD1,ANTXR1,Beta Arrestin,CD34,Cg,CHD4,CHRNA4,CHRNA5,CHRNB2,CHRNB3,CHRNB4,CLIP3,COL18A1,DES,ERRFI1,ESM1,F2RL1,FGF8,FGFR1,GABBR2,HIPK2,MDM2,NCEH1,NRP1,PMAIP1,PTPN13,RASD1,RPA1,RUNX3,SELP,STC1,TRIP6 | 19 | 15 | Psychological Disorders, Cardiovascular Disease, Organismal Injury and Abnormalities |
| 6 | ARL6IP1,ASPM,C8orf4,CAPN2,CAV2,CCNB2,CD47,CDKN3,CEP55,CTSF,DOCK10,EPSTI1,ERG,ESR1,GAS6,HERC5,IFI16,KIF23,KLF9,LGALS3,LGALS3BP,MALL,MAP2K7,N-cor,NCAPG,NRCAM,PDCD6IP,PGR,PRL,RGS3,SLC7A11,SMAD6,TMSB15A,TOP2B,TP53 | 19 | 15 | Cancer, Organismal Injury and Abnormalities, Endocrine System Development and Function |
| 7 | Akt,Calcineurin protein(s),CEBPB,Cg,CXCL1,CXCL8,ERK,estrogen receptor,FBXO32,Fcer1,Fibrinogen,Focal adhesion kinase,GADD45G,Gm-csf,Hdac,HMOX1,Hsp27,Igm,IL6,IRF6,KRT19,Mapk,Mek,N-cor,Nr1h,P38 MAPK,PDGFB,PI3K (family),Pkc(s),PRC1,PTGS2,Ras,SELE,SLC4A7,Vegf | 17 | 14 | Cell-To-Cell Signaling and Interaction, Cellular Movement, Nervous System Development and Function |
| 8 | ACHE,ADORA2B,APP,AQP1,C10orf10,CDCA5,CXCL3,DICER1,ERK,GCLM,IL15RA,LIPG,mir-34,miR-199a-3p (and other miRNAs w/seed CAGUAGU),NEDD4L,PELI2,PF4,PHGDH,RHOU,SCAVENGER receptor CLASS A,SERPINB1,SERTAD4,SIRT6,SLC30A3,SMPD1,STX11,STXBP2,THBD,TNF,TNFRSF6B,TNIP3,TREM1,TRIP6,UACA,ZFP36 | 16 | 13 | Hematological System Development and Function, Tissue Morphology, Cellular Compromise |
| 9 | ABCA1,ADAM15,AGT,ALOX15,CCR6,CD200,CHRNA1,CSNK1A1,FABP4,GAB2,GBP4,GNG7,HAMP,HIF1A,HIST1H2BK,ID3,IL2,IL13,ITGA9,ITGB1,KCNH2,KHDRBS3,KIAA0430,LPXN,MAPK13,NOTCH4,PDK1,SHH,SMARCA4,SPOCD1,STC2,TCR,TEC,TLR6,TPT1 | 12 | 11 | Cellular Movement, Immune Cell Trafficking, Cellular Function and Maintenance |
| 10 | ACSS1,ANLN,CCNA2,CENPC,CEP97,CITED2,CKS2,DDX11,DNTTIP1,E2F4,E2F8,HIST1H2AB,HIST1H2BB,HIST1H4A,KIF23,KIF20A,LIN28A,MAD2L1,MFGE8,mir-132, NDC80, NGFR, NUPR1, ORC1, PARPBP, PCM1,PCNA,RFC3,RPA1,SAMHD1,SPC24,SPC25,TFDP1,TICRR,TP63 | 8 | 8 | Cell Cycle, DNA Replication, Recombination, and Repair, Cellular Assembly and Organization |
| 11 | NUFIP1,SNORD13 | 2 | 1 | RNA Post-Transcriptional Modification, Cancer, Organismal Injury and Abnormalities |
| 12 | ASNA1,CAMLG | 2 | 1 | Cellular Function and Maintenance, Humoral Immune Response, Behavior |
| 13 | CIRBP,WT1 | 2 | 1 | Cell Death and Survival, Cancer, Embryonic Development |
| 14 | FAM46A,PAF1 | 2 | 1 | Cell Cycle, Cellular Assembly and Organization, DNA Replication, Recombination, and Repair |
| 15 | SEMA3G,TCF3 | 2 | 1 | Cellular Development, Hematopoiesis, Tissue Development |
| 16 | RORA,SEMA3F | 2 | 1 | Cell Morphology, Cardiovascular System Development and Function, Nervous System Development and Function |
| 17 | ADAMTS9,mir-29 | 2 | 1 | Cancer, Organismal Injury and Abnormalities, Reproductive System Disease |
| 18 | GPC1,TNFSF13 | 2 | 1 | Cardiovascular System Development and Function, Cell Cycle, Small Molecule Biochemistry |
| 19 | SIAH1,SNCAIP | 2 | 1 | Cell-To-Cell Signaling and Interaction, Drug Metabolism, Molecular Transport |
| 20 | BATF3,DNMT3B | 2 | 1 | DNA Replication, Recombination, and Repair, Developmental Disorder, Gastrointestinal Disease |
| 21 | GGA2,RABEP1 | 2 | 1 | Cell Morphology, Cellular Assembly and Organization, Cellular Function and Maintenance |
| 22 | LARP1,RPL29 | 2 | 1 | Cell Morphology, Cellular Function and Maintenance, Gene Expression |
| 23 | CENPJ,CEP135,SASS6 | 1 | 1 | Cell Cycle, Cellular Assembly and Organization, DNA Replication, Recombination, and Repair |
| 24 | AHI1,LMCD1,SRF | 1 | 1 | Cell Morphology, Cellular Assembly and Organization, Cellular Function and Maintenance |
