# Supplementary material for: PDGFR-alpha inhibits melanoma growth via CXCL10/IP-10: a multi-omics approach
Source: Oncotarget. 2016 Oct 13;7(47):77257–75. doi: 10.18632/oncotarget.12629 (PMC5363585; doi:10.18632/oncotarget.12629)
Supplement: Supplementary file 6 [file oncotarget-07-77257-s006.docx]

**Supplementary Table S5B. Ingenuity Pathway Analysis of differentially expressed transcripts in SKMel-28 cells (networks). In capitol letters genes from the imput list.**

| **ID** | **Molecules in Network** | **Score** | **Focus Molecules** | **Top Diseases and Functions** |
| --- | --- | --- | --- | --- |
| 1 | CCL3,CCL3L1,CXCL9,CXCL10,Fcer1,IDO1,IFIH1,IFIT1,IFIT2,IFIT3,IFITM3,IFN Beta,Ifn gamma,IFN type 1,Iga,IL12 (complex),Immunoglobulin,Interferon alpha,IRF1,IRF9,ISG15,MLKL,MX1,NFkB (complex),Oas,OAS1,OAS2,RARRES3,RSAD2,SOCS1,SOCS3,TCR,Tlr,TNFSF13B,USP18 | 43 | 23 | Antimicrobial Response, Inflammatory Response, Infectious Diseases |
| 2 | CXCL10,DHX58,EPSTI1,EZH2,GBP2,HERC5,IFI16,IFI35,IFI44,IFI44L,IFIH1,IFIT1,IFN Beta,IFNA2,IFNL1,IRF9,MAP2K4,MAP2K7,MAP3K7,NCOA7,PARP,PARP14,PRL,RARRES3,RSAD2,SAA1,SAMD9,SAMD9L,SP110,TAB1,TMEM173,TRIM25,TRIM38,USP18,XAF1 | 35 | 20 | Infectious Diseases, Antimicrobial Response, Inflammatory Response |
| 3 | ACPP,ACTA2,ACTG2,CCL3L3,CD44,FSH,GBP4,GK,HERC5,HIF1A,HIST1H2BK,HSPA6,HSPA1A/HSPA1B,IFI27,IFIT2,IFIT3,IFITM2,INHBA,LAP3,Lh,MAFB,MAPK1,MX2,NEXN,NR3C1,OASL,PAF1,PARP12,PPRC1,PSIP1,RUNX2,SPRR2D,SPRR2F,TCR,ZC3HAV1 | 35 | 20 | Cancer, Organismal Injury and Abnormalities, Renal and Urological Disease |
| 4 | Akt,CD3,CEBPD,Cg,EGR2,ERK,ERK1/2,Focal adhesion kinase,FST,GBP1,GCLM,Growth hormone,HIST1H4C,Histone h3,Histone h4,HMOX1,ICAM1,IFITM1,IgG,IL1B,ITK,Jnk,MAF,Mek,P38 MAPK,PDGF BB,PI3K (complex),PI3K (family),PMAIP1,RASGRP3,SERPINH1,TAP1,TNFSF10,TXNIP,ZFP36 | 33 | 19 | Cellular Development, Hematological System Development and Function, Cellular Growth and Proliferation |
| 5 | RDH5,S100A6 | 2 | 1 | Lipid Metabolism, Small Molecule Biochemistry, Vitamin and Mineral Metabolism |
| 6 | LYPLA2,miR-296-5p (miRNAs w/seed GGGCCCC) | 2 | 1 | Carbohydrate Metabolism, Hematological Disease, Immunological Disease |
| 7 | GABARAPL1,HDAC6 | 2 | 1 | Cell-To-Cell Signaling and Interaction, Cellular Assembly and Organization, Cellular Function and Maintenance |
| 8 | IGFBP2,INO80B | 2 | 1 | Tissue Morphology, Cancer, Cell Morphology |
| 9 | NCL,SNORD3A,SSB | 2 | 1 | Gene Expression, Infectious Diseases, Organismal Development |
| 10 | KHSRP,PDCD6,PLSCR4 | 2 | 1 | Cellular Assembly and Organization, Cell Cycle, RNA Damage and Repair |
| 11 | BSG,NCSTN,NT5C3A | 2 | 1 | Cardiovascular Disease, Cell Death and Survival, Connective Tissue Disorders |
| 12 | CENPJ,CEP135,SASS6 | 2 | 1 | Cell Cycle, Cellular Assembly and Organization, DNA Replication, Recombination, and Repair |
| 13 | CHRNA4,CHRNA5,CHRNA6,CHRNB2,CHRNB3,CHRNB4 | 1 | 1 | Organismal Injury and Abnormalities, Inflammatory Disease, Inflammatory Response |
