# Supplementary material for: PDGFR-alpha inhibits melanoma growth via CXCL10/IP-10: a multi-omics approach
Source: Oncotarget. 2016 Oct 13;7(47):77257–75. doi: 10.18632/oncotarget.12629 (PMC5363585; doi:10.18632/oncotarget.12629)
Supplement: Supplementary file 7 [file oncotarget-07-77257-s007.docx]

**Supplementary Table S6. Differentially expressed mRNAs targeted by differentially expressed miRNAs (high prediction/experimental validation) in HUVEC cells.**

| **miRNA ID** | **Symbol** | **p-value** | **miRNA**  **Fold-Change** | **Source** | **Confidence** | **IPA**  **target ID** | **Gene Symbol** | **mRNA**  **Fold-Change** | **Pathway** |
| --- | --- | --- | --- | --- | --- | --- | --- | --- | --- |
|  |  |  |  |  |  |  |  |  |  |
| hsa-miR-503 | miR-503-5p (miRNAs w/seed AGCAGCG) | 0.01610 | -6.39 | TargetScan Human | High (predicted) | SPRED1 | SPRED1 | 1.53 |  |
| hsa-miR-503 | miR-503-5p (miRNAs w/seed AGCAGCG) | 0.01610 | -6.39 | TargetScan Human | High (predicted) | ELMOD1 | ELMOD1 | 2.23 |  |
| hsa-miR-503 | miR-503-5p (miRNAs w/seed AGCAGCG) | 0.01610 | -6.39 | TargetScan Human | High (predicted) | ADAMTS1 | ADAMTS1 | 2.55 | Axonal Guidance Signaling |
| **hsa-miR-503** | **miR-503-5p (miRNAs w/seed AGCAGCG)** | **0.01610** | **-6.39** | **TargetScan Human** | **High (predicted)** | **CXCL10** | **CXCL10** | **9.02** | **Agranulocyte Adhesion and Diapedesis,Communication between Innate and Adaptive Immune Cells,Granulocyte Adhesion and Diapedesis,IL-17 Signaling,IL-17A Signaling in Gastric Cells,Pathogenesis of Multiple Sclerosis,Role of Hypercytokinemia/hyperchemokinemia in the Pathogenesis of Influenza,Role of IL-17F in Allergic Inflammatory Airway Diseases,Role of MAPK Signaling in the Pathogenesis of Influenza,Thyroid Cancer Signaling,VDR/RXR Activation** |
| hsa-miR-324-5p | miR-324-5p (miRNAs w/seed GCAUCCC) | 0.03990 | -3.94 | TargetScan Human | High (predicted) | TBC1D2 | TBC1D2 | 1.60 |  |
| hsa-miR-324-5p | miR-324-5p (miRNAs w/seed GCAUCCC) | 0.03990 | -3.94 | TargetScan Human | High (predicted) | SASS6 | SASS6 | 1.69 |  |
| hsa-miR-342-3p | miR-342-3p (miRNAs w/seed CUCACAC) | 0.00437 | -3.31 | TargetScan Human | High (predicted) | PTGER4 | PTGER4 | 1.56 | cAMP-mediated signaling,Colorectal Cancer Metastasis Signaling,Eicosanoid Signaling,G-Protein Coupled Receptor Signaling,Gαs Signaling |
| hsa-miR-342-3p | miR-342-3p (miRNAs w/seed CUCACAC) | 0.00437 | -3.31 | TargetScan Human | High (predicted) | NCOA7 | NCOA7 | 1.58 | Aryl Hydrocarbon Receptor Signaling |
| hsa-miR-342-3p | miR-342-3p (miRNAs w/seed CUCACAC) | 0.00437 | -3.31 | TargetScan Human | High (predicted) | IRF1 | IRF1 | 1.75 | IL-12 Signaling and Production in Macrophages,IL-15 Production,iNOS Signaling,Interferon Signaling,Production of Nitric Oxide and Reactive Oxygen Species in Macrophages,Prolactin Signaling,Retinoic acid Mediated Apoptosis Signaling,Role of PKR in Interferon Induction and Antiviral Response,Type I Diabetes Mellitus Signaling |
| hsa-miR-342-3p | miR-342-3p (miRNAs w/seed CUCACAC) | 0.00437 | -3.31 | TargetScan Human | High (predicted) | SELE | SELE | 2.14 | Agranulocyte Adhesion and Diapedesis,Atherosclerosis Signaling,Glucocorticoid Receptor Signaling,Granulocyte Adhesion and Diapedesis,HMGB1 Signaling,Role of Macrophages, Fibroblasts and Endothelial Cells in Rheumatoid Arthritis |
| hsa-miR-217 | miR-217-5p (and other miRNAs w/seed ACUGCAU) | 0.01260 | -2.60 | TargetScan Human | High (predicted) | NCOA7 | NCOA7 | 1.58 | Aryl Hydrocarbon Receptor Signaling |
| hsa-miR-217 | miR-217-5p (and other miRNAs w/seed ACUGCAU) | 0.01260 | -2.60 | TargetScan Human | High (predicted) | ELMOD1 | ELMOD1 | 2.23 |  |
| hsa-miR-494 | miR-494-3p (miRNAs w/seed GAAACAU) | 0.01850 | -2.47 | miRecords | Experimentally Observed | HMOX1 | HMOX1 | 2.02 | Acute Phase Response Signaling,Antioxidant Action of Vitamin C,Choline Biosynthesis III,Endothelin-1 Signaling,Fcγ Receptor-mediated Phagocytosis in Macrophages and Monocytes,Gαq Signaling,Heme Degradation,IL-10 Signaling,IL-8 Signaling,mTOR Signaling,NRF2-mediated Oxidative Stress Response,Pancreatic Adenocarcinoma Signaling,Phospholipase C Signaling,Phospholipases,Xenobiotic Metabolism Signaling |
| hsa-miR-494 | miR-494-3p (miRNAs w/seed GAAACAU) | 0.01850 | -2.47 | TargetScan Human | High (predicted) | SHISA2 | SHISA2 | 2.22 |  |
| hsa-miR-216a | miR-216a-5p (miRNAs w/seed AAUCUCA) | 0.01120 | -2.20 | TargetScan Human | High (predicted) | KIAA1199 | CEMIP | 2.65 | Chondroitin Sulfate Degradation (Metazoa),Dermatan Sulfate Degradation (Metazoa) |
| hsa-miR-93 | miR-17-5p (and other miRNAs w/seed AAAGUGC) | 0.01910 | -2.11 | TargetScan Human | High (predicted) | SLC4A7 | SLC4A7 | 1.50 |  |
| hsa-miR-93 | miR-17-5p (and other miRNAs w/seed AAAGUGC) | 0.01910 | -2.11 | TargetScan Human,miRecords | Experimentally Observed,High (predicted) | IL8 | CXCL8 | 1.53 | Agranulocyte Adhesion and Diapedesis,Airway Pathology in Chronic Obstructive Pulmonary Disease,Atherosclerosis Signaling,Bladder Cancer Signaling,Communication between Innate and Adaptive Immune Cells,Glucocorticoid Receptor Signaling,Granulocyte Adhesion and Diapedesis,Hematopoiesis from Pluripotent Stem Cells,Hepatic Cholestasis,Hepatic Fibrosis / Hepatic Stellate Cell Activation,HMGB1 Signaling,IL-15 Signaling,IL-17 Signaling,IL-17A Signaling in Gastric Cells,IL-6 Signaling,IL-8 Signaling,Role of Cytokines in Mediating Communication between Immune Cells,Role of Hypercytokinemia/hyperchemokinemia in the Pathogenesis of Influenza,Role of IL-17A in Arthritis,Role of IL-17A in Psoriasis,Role of IL-17F in Allergic Inflammatory Airway Diseases,Role of Macrophages, Fibroblasts and Endothelial Cells in Rheumatoid Arthritis,Role of Pattern Recognition Receptors in Recognition of Bacteria and Viruses,Role of Tissue Factor in Cancer,TREM1 Signaling |
| hsa-miR-93 | miR-17-5p (and other miRNAs w/seed AAAGUGC) | 0.01910 | -2.11 | TargetScan Human | High (predicted) | SPRED1 | SPRED1 | 1.53 |  |
| hsa-miR-24 | miR-24-3p (and other miRNAs w/seed GGCUCAG) | 0.00577 | -2.11 | Ingenuity Expert Findings | Experimentally Observed | PMAIP1 | PMAIP1 | 1.53 | Molecular Mechanisms of Cancer,p53 Signaling |
| hsa-miR-24 | miR-24-3p (and other miRNAs w/seed GGCUCAG) | 0.00577 | -2.11 | TargetScan Human | High (predicted) | PTGER4 | PTGER4 | 1.56 | cAMP-mediated signaling,Colorectal Cancer Metastasis Signaling,Eicosanoid Signaling,G-Protein Coupled Receptor Signaling,Gαs Signaling |
| hsa-miR-24 | miR-24-3p (and other miRNAs w/seed GGCUCAG) | 0.00577 | -2.11 | TargetScan Human,miRecords | Experimentally Observed,Moderate (predicted) | MYC | MYC | 1.59 | Acute Myeloid Leukemia Signaling,Aryl Hydrocarbon Receptor Signaling,Bladder Cancer Signaling,Cell Cycle: G1/S Checkpoint Regulation,Chronic Myeloid Leukemia Signaling,Colorectal Cancer Metastasis Signaling,Endometrial Cancer Signaling,Endothelin-1 Signaling,ErbB2-ErbB3 Signaling,ERK/MAPK Signaling,ERK5 Signaling,Estrogen-mediated S-phase Entry,Glioblastoma Multiforme Signaling,ILK Signaling,Molecular Mechanisms of Cancer,Mouse Embryonic Stem Cell Pluripotency,Myc Mediated Apoptosis Signaling,Neuregulin Signaling,P2Y Purigenic Receptor Signaling Pathway,p38 MAPK Signaling,PDGF Signaling,Polyamine Regulation in Colon Cancer,Prolactin Signaling,Role of Macrophages, Fibroblasts and Endothelial Cells in Rheumatoid Arthritis,Small Cell Lung Cancer Signaling,STAT3 Pathway,Telomerase Signaling,Thrombopoietin Signaling,Thyroid Cancer Signaling,Wnt/β-catenin Signaling |
| hsa-miR-93 | miR-17-5p (and other miRNAs w/seed AAAGUGC) | 0.01910 | -2.11 | TargetScan Human | High (predicted) | GABBR2 | GABBR2 | 1.62 | cAMP-mediated signaling,G-Protein Coupled Receptor Signaling,GABA Receptor Signaling,Gαi Signaling |
| hsa-miR-24 | miR-24-3p (and other miRNAs w/seed GGCUCAG) | 0.00577 | -2.11 | TargetScan Human | High (predicted) | STC2 | STC2 | 1.63 |  |
| hsa-miR-93 | miR-17-5p (and other miRNAs w/seed AAAGUGC) | 0.01910 | -2.11 | TargetScan Human | High (predicted) | BMP2 | BMP2 | 1.66 | Adipogenesis pathway,Axonal Guidance Signaling,Basal Cell Carcinoma Signaling,BMP signaling pathway,Cardiomyocyte Differentiation via BMP Receptors,Factors Promoting Cardiogenesis in Vertebrates,Human Embryonic Stem Cell Pluripotency,ILK Signaling,Molecular Mechanisms of Cancer,NF-κB Signaling,RAR Activation,Retinoate Biosynthesis I,Role of NANOG in Mammalian Embryonic Stem Cell Pluripotency,Role of Osteoblasts, Osteoclasts and Chondrocytes in Rheumatoid Arthritis,TGF-β Signaling |
| hsa-miR-93 | miR-17-5p (and other miRNAs w/seed AAAGUGC) | 0.01910 | -2.11 | TargetScan Human | High (predicted) | AADACL1 | NCEH1 | 1.68 |  |
| hsa-miR-93 | miR-17-5p (and other miRNAs w/seed AAAGUGC) | 0.01910 | -2.11 | TargetScan Human | High (predicted) | IRF1 | IRF1 | 1.75 | IL-12 Signaling and Production in Macrophages,IL-15 Production,iNOS Signaling,Interferon Signaling,Production of Nitric Oxide and Reactive Oxygen Species in Macrophages,Prolactin Signaling,Retinoic acid Mediated Apoptosis Signaling,Role of PKR in Interferon Induction and Antiviral Response,Type I Diabetes Mellitus Signaling |
| hsa-miR-24 | miR-24-3p (and other miRNAs w/seed GGCUCAG) | 0.00577 | -2.11 | TargetScan Human | High (predicted) | IRF1 | IRF1 | 1.75 | IL-12 Signaling and Production in Macrophages,IL-15 Production,iNOS Signaling,Interferon Signaling,Production of Nitric Oxide and Reactive Oxygen Species in Macrophages,Prolactin Signaling,Retinoic acid Mediated Apoptosis Signaling,Role of PKR in Interferon Induction and Antiviral Response,Type I Diabetes Mellitus Signaling |
| hsa-miR-93 | miR-17-5p (and other miRNAs w/seed AAAGUGC) | 0.01910 | -2.11 | TargetScan Human | High (predicted) | STC1 | STC1 | 2.78 |  |
| hsa-miR-93 | miR-17-5p (and other miRNAs w/seed AAAGUGC) | 0.01910 | -2.11 | TargetScan Human | High (predicted) | RASD1 | RASD1 | 3.31 | nNOS Signaling in Neurons |
| hsa-miR-93 | miR-17-5p (and other miRNAs w/seed AAAGUGC) | 0.01910 | -2.11 | TargetScan Human | High (predicted) | HSPA6 | HSPA6 | 7.14 | Aldosterone Signaling in Epithelial Cells,eNOS Signaling,Glucocorticoid Receptor Signaling,Huntington's Disease Signaling,Protein Ubiquitination Pathway,Unfolded protein response |
| hsa-miR-99b | miR-100-5p (and other miRNAs w/seed ACCCGUA) | 0.04390 | -2.10 | TargetScan Human | High (predicted) | TRIB1 | TRIB1 | 2.17 |  |
| hsa-miR-103 | miR-103-3p (and other miRNAs w/seed GCAGCAU) | 0.02130 | -2.05 | TargetScan Human | High (predicted) | SNCG | SNCG | 1.64 |  |
| hsa-miR-140-3p | miR-140-3p (and other miRNAs w/seed ACCACAG) | 0.04520 | -2.04 | TargetScan Human | High (predicted) | PPAP2B | PLPP3 | 2.08 | Triacylglycerol Biosynthesis |
| hsa-miR-130a | miR-130a-3p (and other miRNAs w/seed AGUGCAA) | 0.01310 | -2.00 | TargetScan Human | High (predicted) | SPRED1 | SPRED1 | 1.53 |  |
| hsa-miR-130a | miR-130a-3p (and other miRNAs w/seed AGUGCAA) | 0.01310 | -2.00 | TargetScan Human | High (predicted) | IRF1 | IRF1 | 1.75 | IL-12 Signaling and Production in Macrophages,IL-15 Production,iNOS Signaling,Interferon Signaling,Production of Nitric Oxide and Reactive Oxygen Species in Macrophages,Prolactin Signaling,Retinoic acid Mediated Apoptosis Signaling,Role of PKR in Interferon Induction and Antiviral Response,Type I Diabetes Mellitus Signaling |
| hsa-miR-130a | miR-130a-3p (and other miRNAs w/seed AGUGCAA) | 0.01310 | -2.00 | TargetScan Human | High (predicted) | STC1 | STC1 | 2.78 |  |
| hsa-miR-130a | miR-130a-3p (and other miRNAs w/seed AGUGCAA) | 0.01310 | -2.00 | TargetScan Human | High (predicted) | RASD1 | RASD1 | 3.31 | nNOS Signaling in Neurons |
| hsa-let-7b | let-7a-5p (and other miRNAs w/seed GAGGUAG) | 0.03410 | -1.99 | TargetScan Human | High (predicted) | SLC4A7 | SLC4A7 | 1.50 |  |
| hsa-let-7b | let-7a-5p (and other miRNAs w/seed GAGGUAG) | 0.03410 | -1.99 | TargetScan Human | High (predicted) | PMAIP1 | PMAIP1 | 1.53 | Molecular Mechanisms of Cancer,p53 Signaling |
| hsa-let-7b | let-7a-5p (and other miRNAs w/seed GAGGUAG) | 0.03410 | -1.99 | TargetScan Human | High (predicted) | SEMA3F | SEMA3F | 1.53 | Axonal Guidance Signaling,RhoA Signaling |
| hsa-let-7b | let-7a-5p (and other miRNAs w/seed GAGGUAG) | 0.03410 | -1.99 | Ingenuity Expert Findings,TarBase,miRecords | Experimentally Observed | MYC | MYC | 1.59 | Acute Myeloid Leukemia Signaling,Aryl Hydrocarbon Receptor Signaling,Bladder Cancer Signaling,Cell Cycle: G1/S Checkpoint Regulation,Chronic Myeloid Leukemia Signaling,Colorectal Cancer Metastasis Signaling,Endometrial Cancer Signaling,Endothelin-1 Signaling,ErbB2-ErbB3 Signaling,ERK/MAPK Signaling,ERK5 Signaling,Estrogen-mediated S-phase Entry,Glioblastoma Multiforme Signaling,ILK Signaling,Molecular Mechanisms of Cancer,Mouse Embryonic Stem Cell Pluripotency,Myc Mediated Apoptosis Signaling,Neuregulin Signaling,P2Y Purigenic Receptor Signaling Pathway,p38 MAPK Signaling,PDGF Signaling,Polyamine Regulation in Colon Cancer,Prolactin Signaling,Role of Macrophages, Fibroblasts and Endothelial Cells in Rheumatoid Arthritis,Small Cell Lung Cancer Signaling,STAT3 Pathway,Telomerase Signaling,Thrombopoietin Signaling,Thyroid Cancer Signaling,Wnt/β-catenin Signaling |
| hsa-let-7b | let-7a-5p (and other miRNAs w/seed GAGGUAG) | 0.03410 | -1.99 | miRecords | Experimentally Observed | KRT19 | KRT19 | 1.61 |  |
| hsa-let-7b | let-7a-5p (and other miRNAs w/seed GAGGUAG) | 0.03410 | -1.99 | TargetScan Human | High (predicted) | GABBR2 | GABBR2 | 1.62 | cAMP-mediated signaling,G-Protein Coupled Receptor Signaling,GABA Receptor Signaling,Gαi Signaling |
| hsa-let-7b | let-7a-5p (and other miRNAs w/seed GAGGUAG) | 0.03410 | -1.99 | miRecords | Experimentally Observed | HMOX1 | HMOX1 | 2.02 | Acute Phase Response Signaling,Antioxidant Action of Vitamin C,Choline Biosynthesis III,Endothelin-1 Signaling,Fcγ Receptor-mediated Phagocytosis in Macrophages and Monocytes,Gαq Signaling,Heme Degradation,IL-10 Signaling,IL-8 Signaling,mTOR Signaling,NRF2-mediated Oxidative Stress Response,Pancreatic Adenocarcinoma Signaling,Phospholipase C Signaling,Phospholipases,Xenobiotic Metabolism Signaling |
| hsa-let-7b | let-7a-5p (and other miRNAs w/seed GAGGUAG) | 0.03410 | -1.99 | TargetScan Human | High (predicted) | TRIB1 | TRIB1 | 2.17 |  |
| hsa-let-7b | let-7a-5p (and other miRNAs w/seed GAGGUAG) | 0.03410 | -1.99 | TargetScan Human | High (predicted) | ADAMTS1 | ADAMTS1 | 2.55 | Axonal Guidance Signaling |
| hsa-let-7b | let-7a-5p (and other miRNAs w/seed GAGGUAG) | 0.03410 | -1.99 | TarBase | Experimentally Observed | PTGS2 | PTGS2 | 2.56 | CD40 Signaling,Cholecystokinin/Gastrin-mediated Signaling,Colorectal Cancer Metastasis Signaling,Corticotropin Releasing Hormone Signaling,Eicosanoid Signaling,Endothelin-1 Signaling,Fatty Acid α-oxidation,Glucocorticoid Receptor Signaling,HGF Signaling,IL-17 Signaling,IL-8 Signaling,ILK Signaling,LXR/RXR Activation,MIF Regulation of Innate Immunity,MIF-mediated Glucocorticoid Regulation,Ovarian Cancer Signaling,Pancreatic Adenocarcinoma Signaling,PI3K/AKT Signaling,PPAR Signaling,Prostanoid Biosynthesis,Protein Kinase A Signaling,Role of IL-17A in Arthritis,Role of MAPK Signaling in the Pathogenesis of Influenza,Small Cell Lung Cancer Signaling |
| hsa-let-7b | let-7a-5p (and other miRNAs w/seed GAGGUAG) | 0.03410 | -1.99 | TargetScan Human | High (predicted) | CEBPD | CEBPD | 2.61 | Adipogenesis pathway,IL-17A Signaling in Fibroblasts,Role of Macrophages, Fibroblasts and Endothelial Cells in Rheumatoid Arthritis,Unfolded protein response |
| hsa-let-7b | let-7a-5p (and other miRNAs w/seed GAGGUAG) | 0.03410 | -1.99 | TargetScan Human | High (predicted) | IL6 | IL6 | 3.33 | Activation of IRF by Cytosolic Pattern Recognition Receptors,Acute Phase Response Signaling,Altered T Cell and B Cell Signaling in Rheumatoid Arthritis,Aryl Hydrocarbon Receptor Signaling,Atherosclerosis Signaling,Cardiac Hypertrophy Signaling,Colorectal Cancer Metastasis Signaling,Communication between Innate and Adaptive Immune Cells,Crosstalk between Dendritic Cells and Natural Killer Cells,Dendritic Cell Maturation,Differential Regulation of Cytokine Production in Macrophages and T Helper Cells by IL-17A and IL-17F,Glucocorticoid Receptor Signaling,Graft-versus-Host Disease Signaling,Hematopoiesis from Pluripotent Stem Cells,Hepatic Cholestasis,Hepatic Fibrosis / Hepatic Stellate Cell Activation,HGF Signaling,HMGB1 Signaling,IL-10 Signaling,IL-15 Production,IL-15 Signaling,IL-17 Signaling,IL-17A Signaling in Airway Cells,IL-17A Signaling in Fibroblasts,IL-6 Signaling,JAK/Stat Signaling,LXR/RXR Activation,PPARα/RXRα Activation,PXR/RXR Activation,Role of Cytokines in Mediating Communication between Immune Cells,Role of Hypercytokinemia/hyperchemokinemia in the Pathogenesis of Influenza,Role of IL-17F in Allergic Inflammatory Airway Diseases,Role of JAK family kinases in IL-6-type Cytokine Signaling,Role of Macrophages, Fibroblasts and Endothelial Cells in Rheumatoid Arthritis,Role of NFAT in Cardiac Hypertrophy,Role of Osteoblasts, Osteoclasts and Chondrocytes in Rheumatoid Arthritis,Role of Pattern Recognition Receptors in Recognition of Bacteria and Viruses,Systemic Lupus Erythematosus Signaling,T Helper Cell Differentiation,TREM1 Signaling,Xenobiotic Metabolism Signaling |
| hsa-miR-29b | miR-29b-3p (and other miRNAs w/seed AGCACCA) | 0.00972 | -1.96 | TargetScan Human | High (predicted) | TMEM163 | TMEM163 | 1.51 |  |
| hsa-miR-29b | miR-29b-3p (and other miRNAs w/seed AGCACCA) | 0.00972 | -1.96 | Ingenuity Expert Findings,TargetScan Human | Experimentally Observed,High (predicted) | GPR37 | GPR37 | 1.54 | Cellular Effects of Sildenafil (Viagra),GABA Receptor Signaling,Neuropathic Pain Signaling In Dorsal Horn Neurons,Parkinson's Signaling |
| hsa-miR-29b | miR-29b-3p (and other miRNAs w/seed AGCACCA) | 0.00972 | -1.96 | TargetScan Human | High (predicted) | IL1RL1 | IL1RL1 | 1.69 | Granulocyte Adhesion and Diapedesis,Hepatic Cholestasis,Hepatic Fibrosis / Hepatic Stellate Cell Activation,IL-10 Signaling,IL-6 Signaling,LPS/IL-1 Mediated Inhibition of RXR Function,LXR/RXR Activation,p38 MAPK Signaling,PPAR Signaling,PPARα/RXRα Activation,Role of Macrophages, Fibroblasts and Endothelial Cells in Rheumatoid Arthritis,Role of Osteoblasts, Osteoclasts and Chondrocytes in Rheumatoid Arthritis,Toll-like Receptor Signaling,TREM1 Signaling,VDR/RXR Activation |
| hsa-miR-29b | miR-29b-3p (and other miRNAs w/seed AGCACCA) | 0.00972 | -1.96 | miRecords | Experimentally Observed | TGFB3 | TGFB3 | 1.76 | Antiproliferative Role of TOB in T Cell Signaling,Aryl Hydrocarbon Receptor Signaling,Cardiac Hypertrophy Signaling,Cell Cycle: G1/S Checkpoint Regulation,Chronic Myeloid Leukemia Signaling,Colorectal Cancer Metastasis Signaling,Cyclins and Cell Cycle Regulation,Factors Promoting Cardiogenesis in Vertebrates,Germ Cell-Sertoli Cell Junction Signaling,Glucocorticoid Receptor Signaling,Hepatic Cholestasis,Hepatic Fibrosis / Hepatic Stellate Cell Activation,HMGB1 Signaling,Human Embryonic Stem Cell Pluripotency,IL-12 Signaling and Production in Macrophages,Molecular Mechanisms of Cancer,p38 MAPK Signaling,Pancreatic Adenocarcinoma Signaling,PPARα/RXRα Activation,Protein Kinase A Signaling,RAR Activation,Regulation of IL-2 Expression in Activated and Anergic T Lymphocytes,Regulation of the Epithelial-Mesenchymal Transition Pathway,Role of NFAT in Cardiac Hypertrophy,Role of Pattern Recognition Receptors in Recognition of Bacteria and Viruses,Sertoli Cell-Sertoli Cell Junction Signaling,TGF-β Signaling,Tight Junction Signaling,Wnt/β-catenin Signaling |
| hsa-miR-29b | miR-29b-3p (and other miRNAs w/seed AGCACCA) | 0.00972 | -1.96 | TargetScan Human | High (predicted) | KIAA1199 | CEMIP | 2.65 | Chondroitin Sulfate Degradation (Metazoa),Dermatan Sulfate Degradation (Metazoa) |
| hsa-miR-29b | miR-29b-3p (and other miRNAs w/seed AGCACCA) | 0.00972 | -1.96 | TargetScan Human | High (predicted) | ADAMTS9 | ADAMTS9 | 2.78 | Axonal Guidance Signaling |
| hsa-miR-29b | miR-29b-3p (and other miRNAs w/seed AGCACCA) | 0.00972 | -1.96 | TargetScan Human | High (predicted) | ZFP36 | ZFP36 | 2.91 |  |
| hsa-miR-376c | miR-376c-3p (and other miRNAs w/seed ACAUAGA) | 0.04010 | -1.96 | TargetScan Human | High (predicted) | SLC7A11 | SLC7A11 | 1.55 |  |
| hsa-miR-376c | miR-376c-3p (and other miRNAs w/seed ACAUAGA) | 0.04010 | -1.96 | TargetScan Human | High (predicted) | NAP1L5 | NAP1L5 | 1.56 |  |
| hsa-miR-376c | miR-376c-3p (and other miRNAs w/seed ACAUAGA) | 0.04010 | -1.96 | TargetScan Human | High (predicted) | CDH11 | CDH11 | 1.62 | Gα12/13 Signaling,RhoGDI Signaling,Signaling by Rho Family GTPases |
| hsa-miR-376c | miR-376c-3p (and other miRNAs w/seed ACAUAGA) | 0.04010 | -1.96 | TargetScan Human | High (predicted) | PPAP2B | PLPP3 | 2.08 | Triacylglycerol Biosynthesis |
| hsa-miR-221 | miR-221-3p (and other miRNAs w/seed GCUACAU) | 0.01610 | -1.93 | TargetScan Human,miRecords | Experimentally Observed,Moderate (predicted) | MMP1 | MMP1 | 1.53 | Agranulocyte Adhesion and Diapedesis,Airway Pathology in Chronic Obstructive Pulmonary Disease,Atherosclerosis Signaling,Bladder Cancer Signaling,Colorectal Cancer Metastasis Signaling,Glucocorticoid Receptor Signaling,Granulocyte Adhesion and Diapedesis,Hepatic Fibrosis / Hepatic Stellate Cell Activation,HIF1α Signaling,IL-17A Signaling in Fibroblasts,Inhibition of Matrix Metalloproteases,Leukocyte Extravasation Signaling,Oncostatin M Signaling,RAR Activation,Role of IL-17A in Arthritis,Role of Macrophages, Fibroblasts and Endothelial Cells in Rheumatoid Arthritis,Role of Osteoblasts, Osteoclasts and Chondrocytes in Rheumatoid Arthritis,Role of Tissue Factor in Cancer |
| hsa-miR-221 | miR-221-3p (and other miRNAs w/seed GCUACAU) | 0.01610 | -1.93 | TargetScan Human | High (predicted) | NAP1L5 | NAP1L5 | 1.56 |  |
| hsa-miR-221 | miR-221-3p (and other miRNAs w/seed GCUACAU) | 0.01610 | -1.93 | TargetScan Human | High (predicted) | SOCS1 | SOCS1 | 1.78 | Acute Phase Response Signaling,Erythropoietin Signaling,Growth Hormone Signaling,IGF-1 Signaling,IL-2 Signaling,IL-4 Signaling,IL-6 Signaling,Interferon Signaling,JAK/Stat Signaling,Prolactin Signaling,Role of JAK family kinases in IL-6-type Cytokine Signaling,Role of JAK1 and JAK3 in γc Cytokine Signaling,Role of JAK1, JAK2 and TYK2 in Interferon Signaling,Role of JAK2 in Hormone-like Cytokine Signaling,Role of Macrophages, Fibroblasts and Endothelial Cells in Rheumatoid Arthritis,STAT3 Pathway,Type I Diabetes Mellitus Signaling,Type II Diabetes Mellitus Signaling |
| hsa-miR-221 | miR-221-3p (and other miRNAs w/seed GCUACAU) | 0.01610 | -1.93 | TargetScan Human | High (predicted) | SOCS3 | SOCS3 | 1.84 | 3-phosphoinositide Biosynthesis,3-phosphoinositide Degradation,Acute Phase Response Signaling,D-myo-inositol (1,4,5,6)-Tetrakisphosphate Biosynthesis,D-myo-inositol (3,4,5,6)-tetrakisphosphate Biosynthesis,D-myo-inositol-5-phosphate Metabolism,Erythropoietin Signaling,Growth Hormone Signaling,IGF-1 Signaling,IL-10 Signaling,IL-22 Signaling,IL-6 Signaling,IL-9 Signaling,Insulin Receptor Signaling,JAK/Stat Signaling,Leptin Signaling in Obesity,Prolactin Signaling,Role of JAK family kinases in IL-6-type Cytokine Signaling,Role of JAK1 and JAK3 in γc Cytokine Signaling,Role of JAK2 in Hormone-like Cytokine Signaling,Role of Macrophages, Fibroblasts and Endothelial Cells in Rheumatoid Arthritis,STAT3 Pathway,Superpathway of Inositol Phosphate Compounds,Type I Diabetes Mellitus Signaling,Type II Diabetes Mellitus Signaling |
| hsa-miR-221 | miR-221-3p (and other miRNAs w/seed GCUACAU) | 0.01610 | -1.93 | TargetScan Human | High (predicted) | ZFP36 | ZFP36 | 2.91 |  |
| hsa-miR-181b | miR-181a-5p (and other miRNAs w/seed ACAUUCA) | 0.03700 | -1.86 | TargetScan Human | High (predicted) | SLC7A11 | SLC7A11 | 1.55 |  |
| hsa-miR-181b | miR-181a-5p (and other miRNAs w/seed ACAUUCA) | 0.03700 | -1.86 | TargetScan Human | High (predicted) | NAP1L5 | NAP1L5 | 1.56 |  |
| hsa-miR-181b | miR-181a-5p (and other miRNAs w/seed ACAUUCA) | 0.03700 | -1.86 | TargetScan Human | High (predicted) | STC2 | STC2 | 1.63 |  |
| hsa-miR-181b | miR-181a-5p (and other miRNAs w/seed ACAUUCA) | 0.03700 | -1.86 | TargetScan Human | High (predicted) | SPP1 | SPP1 | 1.82 | Altered T Cell and B Cell Signaling in Rheumatoid Arthritis,Role of Oct4 in Mammalian Embryonic Stem Cell Pluripotency,Role of Osteoblasts, Osteoclasts and Chondrocytes in Rheumatoid Arthritis,VDR/RXR Activation |
| hsa-miR-181b | miR-181a-5p (and other miRNAs w/seed ACAUUCA) | 0.03700 | -1.86 | TargetScan Human | High (predicted) | SOCS3 | SOCS3 | 1.84 | 3-phosphoinositide Biosynthesis,3-phosphoinositide Degradation,Acute Phase Response Signaling,D-myo-inositol (1,4,5,6)-Tetrakisphosphate Biosynthesis,D-myo-inositol (3,4,5,6)-tetrakisphosphate Biosynthesis,D-myo-inositol-5-phosphate Metabolism,Erythropoietin Signaling,Growth Hormone Signaling,IGF-1 Signaling,IL-10 Signaling,IL-22 Signaling,IL-6 Signaling,IL-9 Signaling,Insulin Receptor Signaling,JAK/Stat Signaling,Leptin Signaling in Obesity,Prolactin Signaling,Role of JAK family kinases in IL-6-type Cytokine Signaling,Role of JAK1 and JAK3 in γc Cytokine Signaling,Role of JAK2 in Hormone-like Cytokine Signaling,Role of Macrophages, Fibroblasts and Endothelial Cells in Rheumatoid Arthritis,STAT3 Pathway,Superpathway of Inositol Phosphate Compounds,Type I Diabetes Mellitus Signaling,Type II Diabetes Mellitus Signaling |
| hsa-miR-181b | miR-181a-5p (and other miRNAs w/seed ACAUUCA) | 0.03700 | -1.86 | TargetScan Human | High (predicted) | DNAJB1 | DNAJB1 | 1.96 | Aldosterone Signaling in Epithelial Cells,Androgen Signaling,Huntington's Disease Signaling,NRF2-mediated Oxidative Stress Response,Protein Ubiquitination Pathway |
| hsa-miR-181b | miR-181a-5p (and other miRNAs w/seed ACAUUCA) | 0.03700 | -1.86 | TargetScan Human | High (predicted) | PPAP2B | PLPP3 | 2.08 | Triacylglycerol Biosynthesis |
| hsa-miR-181b | miR-181a-5p (and other miRNAs w/seed ACAUUCA) | 0.03700 | -1.86 | TargetScan Human | High (predicted) | ADAMTS1 | ADAMTS1 | 2.55 | Axonal Guidance Signaling |
| hsa-miR-181b | miR-181a-5p (and other miRNAs w/seed ACAUUCA) | 0.03700 | -1.86 | TargetScan Human | High (predicted) | STC1 | STC1 | 2.78 |  |
| hsa-miR-181b | miR-181a-5p (and other miRNAs w/seed ACAUUCA) | 0.03700 | -1.86 | TargetScan Human | High (predicted) | CCL8 | CCL8 | 13.10 | Agranulocyte Adhesion and Diapedesis,Granulocyte Adhesion and Diapedesis |
| hsa-miR-125a-5p | miR-125b-5p (and other miRNAs w/seed CCCUGAG) | 0.04930 | -1.84 | TargetScan Human | High (predicted) | FAM46A | FAM46A | 1.58 |  |
| hsa-miR-125a-5p | miR-125b-5p (and other miRNAs w/seed CCCUGAG) | 0.04930 | -1.84 | miRecords | Experimentally Observed | KRT19 | KRT19 | 1.61 |  |
| hsa-miR-125a-5p | miR-125b-5p (and other miRNAs w/seed CCCUGAG) | 0.04930 | -1.84 | miRecords | Experimentally Observed | ADAMTS1 | ADAMTS1 | 2.55 | Axonal Guidance Signaling |
| hsa-miR-34a | miR-34a-5p (and other miRNAs w/seed GGCAGUG) | 0.03260 | -1.81 | TargetScan Human | High (predicted) | SLC4A7 | SLC4A7 | 1.50 |  |
| hsa-miR-34a | miR-34a-5p (and other miRNAs w/seed GGCAGUG) | 0.03260 | -1.81 | TargetScan Human | High (predicted) | SPRED1 | SPRED1 | 1.53 |  |
| hsa-miR-34a | miR-34a-5p (and other miRNAs w/seed GGCAGUG) | 0.03260 | -1.81 | TargetScan Human | High (predicted) | NAP1L5 | NAP1L5 | 1.56 |  |
| hsa-miR-34a | miR-34a-5p (and other miRNAs w/seed GGCAGUG) | 0.03260 | -1.81 | TargetScan Human | High (predicted) | FAM46A | FAM46A | 1.58 |  |
| hsa-miR-34a | miR-34a-5p (and other miRNAs w/seed GGCAGUG) | 0.03260 | -1.81 | TargetScan Human | High (predicted) | FAM107A | FAM107A | 1.59 |  |
| hsa-miR-34a | miR-34a-5p (and other miRNAs w/seed GGCAGUG) | 0.03260 | -1.81 | miRecords | Experimentally Observed | MYC | MYC | 1.59 | Acute Myeloid Leukemia Signaling,Aryl Hydrocarbon Receptor Signaling,Bladder Cancer Signaling,Cell Cycle: G1/S Checkpoint Regulation,Chronic Myeloid Leukemia Signaling,Colorectal Cancer Metastasis Signaling,Endometrial Cancer Signaling,Endothelin-1 Signaling,ErbB2-ErbB3 Signaling,ERK/MAPK Signaling,ERK5 Signaling,Estrogen-mediated S-phase Entry,Glioblastoma Multiforme Signaling,ILK Signaling,Molecular Mechanisms of Cancer,Mouse Embryonic Stem Cell Pluripotency,Myc Mediated Apoptosis Signaling,Neuregulin Signaling,P2Y Purigenic Receptor Signaling Pathway,p38 MAPK Signaling,PDGF Signaling,Polyamine Regulation in Colon Cancer,Prolactin Signaling,Role of Macrophages, Fibroblasts and Endothelial Cells in Rheumatoid Arthritis,Small Cell Lung Cancer Signaling,STAT3 Pathway,Telomerase Signaling,Thrombopoietin Signaling,Thyroid Cancer Signaling,Wnt/β-catenin Signaling |
| hsa-miR-34a | miR-34a-5p (and other miRNAs w/seed GGCAGUG) | 0.03260 | -1.81 | TargetScan Human | High (predicted) | GABBR2 | GABBR2 | 1.62 | cAMP-mediated signaling,G-Protein Coupled Receptor Signaling,GABA Receptor Signaling,Gαi Signaling |
| hsa-miR-34a | miR-34a-5p (and other miRNAs w/seed GGCAGUG) | 0.03260 | -1.81 | TargetScan Human | High (predicted) | AADACL1 | NCEH1 | 1.68 |  |
| hsa-miR-34a | miR-34a-5p (and other miRNAs w/seed GGCAGUG) | 0.03260 | -1.81 | TargetScan Human | High (predicted) | IRF1 | IRF1 | 1.75 | IL-12 Signaling and Production in Macrophages,IL-15 Production,iNOS Signaling,Interferon Signaling,Production of Nitric Oxide and Reactive Oxygen Species in Macrophages,Prolactin Signaling,Retinoic acid Mediated Apoptosis Signaling,Role of PKR in Interferon Induction and Antiviral Response,Type I Diabetes Mellitus Signaling |
| hsa-miR-34a | miR-34a-5p (and other miRNAs w/seed GGCAGUG) | 0.03260 | -1.81 | TargetScan Human | High (predicted) | DNAJB1 | DNAJB1 | 1.96 | Aldosterone Signaling in Epithelial Cells,Androgen Signaling,Huntington's Disease Signaling,NRF2-mediated Oxidative Stress Response,Protein Ubiquitination Pathway |
| hsa-miR-34a | miR-34a-5p (and other miRNAs w/seed GGCAGUG) | 0.03260 | -1.81 | TargetScan Human | High (predicted) | ELMOD1 | ELMOD1 | 2.23 |  |
| hsa-miR-34a | miR-34a-5p (and other miRNAs w/seed GGCAGUG) | 0.03260 | -1.81 | TargetScan Human | High (predicted) | HSPA1A | HSPA1A/HSPA1B | 3.86 | Aldosterone Signaling in Epithelial Cells,eNOS Signaling,Glucocorticoid Receptor Signaling,Huntington's Disease Signaling,Protein Ubiquitination Pathway,Unfolded protein response |
| hsa-miR-27a | miR-27a-3p (and other miRNAs w/seed UCACAGU) | 0.04050 | -1.78 | Ingenuity Expert Findings | Experimentally Observed | PMAIP1 | PMAIP1 | 1.53 | Molecular Mechanisms of Cancer,p53 Signaling |
| hsa-miR-27a | miR-27a-3p (and other miRNAs w/seed UCACAGU) | 0.04050 | -1.78 | TargetScan Human | High (predicted) | SLC7A11 | SLC7A11 | 1.55 |  |
| hsa-miR-27a | miR-27a-3p (and other miRNAs w/seed UCACAGU) | 0.04050 | -1.78 | TargetScan Human | High (predicted) | NAP1L5 | NAP1L5 | 1.56 |  |
| hsa-miR-27a | miR-27a-3p (and other miRNAs w/seed UCACAGU) | 0.04050 | -1.78 | TargetScan Human | High (predicted) | NCOA7 | NCOA7 | 1.58 | Aryl Hydrocarbon Receptor Signaling |
| hsa-miR-27a | miR-27a-3p (and other miRNAs w/seed UCACAGU) | 0.04050 | -1.78 | TargetScan Human | High (predicted) | CDH11 | CDH11 | 1.62 | Gα12/13 Signaling,RhoGDI Signaling,Signaling by Rho Family GTPases |
| hsa-miR-27a | miR-27a-3p (and other miRNAs w/seed UCACAGU) | 0.04050 | -1.78 | TargetScan Human | High (predicted) | NRCAM | NRCAM | 1.80 |  |
| hsa-miR-27a | miR-27a-3p (and other miRNAs w/seed UCACAGU) | 0.04050 | -1.78 | TargetScan Human | High (predicted) | PPAP2B | PLPP3 | 2.08 | Triacylglycerol Biosynthesis |
| hsa-miR-27a | miR-27a-3p (and other miRNAs w/seed UCACAGU) | 0.04050 | -1.78 | TargetScan Human | High (predicted) | CSRP2 | CSRP2 | 2.22 |  |
| hsa-miR-27a | miR-27a-3p (and other miRNAs w/seed UCACAGU) | 0.04050 | -1.78 | TargetScan Human | High (predicted) | ELMOD1 | ELMOD1 | 2.23 |  |
| hsa-miR-27a | miR-27a-3p (and other miRNAs w/seed UCACAGU) | 0.04050 | -1.78 | TargetScan Human | High (predicted) | KIAA1199 | CEMIP | 2.65 | Chondroitin Sulfate Degradation (Metazoa),Dermatan Sulfate Degradation (Metazoa) |
| hsa-miR-27a | miR-27a-3p (and other miRNAs w/seed UCACAGU) | 0.04050 | -1.78 | TargetScan Human | High (predicted) | ZFP36 | ZFP36 | 2.91 |  |
| hsa-miR-26a | miR-26a-5p (and other miRNAs w/seed UCAAGUA) | 0.01420 | -1.77 | TargetScan Human | High (predicted) | PTPN13 | PTPN13 | 1.50 | 3-phosphoinositide Biosynthesis,3-phosphoinositide Degradation,D-myo-inositol (1,4,5,6)-Tetrakisphosphate Biosynthesis,D-myo-inositol (3,4,5,6)-tetrakisphosphate Biosynthesis,D-myo-inositol-5-phosphate Metabolism,Ephrin Receptor Signaling,Protein Kinase A Signaling,Superpathway of Inositol Phosphate Compounds |
| hsa-miR-26a | miR-26a-5p (and other miRNAs w/seed UCAAGUA) | 0.01420 | -1.77 | TargetScan Human | High (predicted) | PMAIP1 | PMAIP1 | 1.53 | Molecular Mechanisms of Cancer,p53 Signaling |
| hsa-miR-26a | miR-26a-5p (and other miRNAs w/seed UCAAGUA) | 0.01420 | -1.77 | TargetScan Human | High (predicted) | SLC7A11 | SLC7A11 | 1.55 |  |
| hsa-miR-26a | miR-26a-5p (and other miRNAs w/seed UCAAGUA) | 0.01420 | -1.77 | TargetScan Human | High (predicted) | NAP1L5 | NAP1L5 | 1.56 |  |
| hsa-miR-26a | miR-26a-5p (and other miRNAs w/seed UCAAGUA) | 0.01420 | -1.77 | TargetScan Human | High (predicted) | CDH11 | CDH11 | 1.62 | Gα12/13 Signaling,RhoGDI Signaling,Signaling by Rho Family GTPases |
| hsa-miR-26a | miR-26a-5p (and other miRNAs w/seed UCAAGUA) | 0.01420 | -1.77 | TargetScan Human | High (predicted) | AADACL1 | NCEH1 | 1.68 |  |
| hsa-miR-26a | miR-26a-5p (and other miRNAs w/seed UCAAGUA) | 0.01420 | -1.77 | TargetScan Human | High (predicted) | NAMPT | NAMPT | 2.41 | NAD Biosynthesis III |
| hsa-miR-26a | miR-26a-5p (and other miRNAs w/seed UCAAGUA) | 0.01420 | -1.77 | Ingenuity Expert Findings,TargetScan Human | Experimentally Observed,High (predicted) | PTGS2 | PTGS2 | 2.56 | CD40 Signaling,Cholecystokinin/Gastrin-mediated Signaling,Colorectal Cancer Metastasis Signaling,Corticotropin Releasing Hormone Signaling,Eicosanoid Signaling,Endothelin-1 Signaling,Fatty Acid α-oxidation,Glucocorticoid Receptor Signaling,HGF Signaling,IL-17 Signaling,IL-8 Signaling,ILK Signaling,LXR/RXR Activation,MIF Regulation of Innate Immunity,MIF-mediated Glucocorticoid Regulation,Ovarian Cancer Signaling,Pancreatic Adenocarcinoma Signaling,PI3K/AKT Signaling,PPAR Signaling,Prostanoid Biosynthesis,Protein Kinase A Signaling,Role of IL-17A in Arthritis,Role of MAPK Signaling in the Pathogenesis of Influenza,Small Cell Lung Cancer Signaling |
| hsa-miR-26a | miR-26a-5p (and other miRNAs w/seed UCAAGUA) | 0.01420 | -1.77 | TargetScan Human | High (predicted) | RHOU | RHOU | 2.74 | Actin Nucleation by ARP-WASP Complex,Cardiac Hypertrophy Signaling,Cholecystokinin/Gastrin-mediated Signaling,Colorectal Cancer Metastasis Signaling,CXCR4 Signaling,Germ Cell-Sertoli Cell Junction Signaling,Glioblastoma Multiforme Signaling,Glioma Invasiveness Signaling,Gαq Signaling,HMGB1 Signaling,IL-8 Signaling,ILK Signaling,Integrin Signaling,Molecular Mechanisms of Cancer,mTOR Signaling,phagosome formation,Phospholipase C Signaling,Production of Nitric Oxide and Reactive Oxygen Species in Macrophages,Regulation of Actin-based Motility by Rho,RhoGDI Signaling,Semaphorin Signaling in Neurons,Signaling by Rho Family GTPases,Sphingosine-1-phosphate Signaling,Tec Kinase Signaling,Thrombin Signaling |
| hsa-miR-574-5p | miR-574-5p (miRNAs w/seed GAGUGUG) | 0.04150 | -1.77 | TargetScan Human | High (predicted) | TRIB1 | TRIB1 | 2.17 |  |
| hsa-miR-214 | miR-214-3p (and other miRNAs w/seed CAGCAGG) | 0.03850 | -1.70 | TargetScan Human | High (predicted) | ERRFI1 | ERRFI1 | 1.55 | Neuregulin Signaling |
| hsa-miR-214 | miR-214-3p (and other miRNAs w/seed CAGCAGG) | 0.03850 | -1.70 | TargetScan Human | High (predicted) | CDH11 | CDH11 | 1.62 | Gα12/13 Signaling,RhoGDI Signaling,Signaling by Rho Family GTPases |
| hsa-miR-214 | miR-214-3p (and other miRNAs w/seed CAGCAGG) | 0.03850 | -1.70 | TargetScan Human | High (predicted) | BMP2 | BMP2 | 1.66 | Adipogenesis pathway,Axonal Guidance Signaling,Basal Cell Carcinoma Signaling,BMP signaling pathway,Cardiomyocyte Differentiation via BMP Receptors,Factors Promoting Cardiogenesis in Vertebrates,Human Embryonic Stem Cell Pluripotency,ILK Signaling,Molecular Mechanisms of Cancer,NF-κB Signaling,RAR Activation,Retinoate Biosynthesis I,Role of NANOG in Mammalian Embryonic Stem Cell Pluripotency,Role of Osteoblasts, Osteoclasts and Chondrocytes in Rheumatoid Arthritis,TGF-β Signaling |
| hsa-miR-214 | miR-214-3p (and other miRNAs w/seed CAGCAGG) | 0.03850 | -1.70 | TargetScan Human | High (predicted) | PPAP2B | PLPP3 | 2.08 | Triacylglycerol Biosynthesis |
| hsa-miR-214 | miR-214-3p (and other miRNAs w/seed CAGCAGG) | 0.03850 | -1.70 | TargetScan Human | High (predicted) | PTGS2 | PTGS2 | 2.56 | CD40 Signaling,Cholecystokinin/Gastrin-mediated Signaling,Colorectal Cancer Metastasis Signaling,Corticotropin Releasing Hormone Signaling,Eicosanoid Signaling,Endothelin-1 Signaling,Fatty Acid α-oxidation,Glucocorticoid Receptor Signaling,HGF Signaling,IL-17 Signaling,IL-8 Signaling,ILK Signaling,LXR/RXR Activation,MIF Regulation of Innate Immunity,MIF-mediated Glucocorticoid Regulation,Ovarian Cancer Signaling,Pancreatic Adenocarcinoma Signaling,PI3K/AKT Signaling,PPAR Signaling,Prostanoid Biosynthesis,Protein Kinase A Signaling,Role of IL-17A in Arthritis,Role of MAPK Signaling in the Pathogenesis of Influenza,Small Cell Lung Cancer Signaling |
| hsa-miR-25 | miR-92a-3p (and other miRNAs w/seed AUUGCAC) | 0.03680 | -1.58 | TargetScan Human | High (predicted) | SLC7A11 | SLC7A11 | 1.55 |  |
| hsa-miR-25 | miR-92a-3p (and other miRNAs w/seed AUUGCAC) | 0.03680 | -1.58 | TargetScan Human | High (predicted) | PTGER4 | PTGER4 | 1.56 | cAMP-mediated signaling,Colorectal Cancer Metastasis Signaling,Eicosanoid Signaling,G-Protein Coupled Receptor Signaling,Gαs Signaling |
| hsa-miR-25 | miR-92a-3p (and other miRNAs w/seed AUUGCAC) | 0.03680 | -1.58 | TargetScan Human | High (predicted) | HES2 | HES2 | 1.59 |  |
| hsa-miR-25 | miR-92a-3p (and other miRNAs w/seed AUUGCAC) | 0.03680 | -1.58 | TargetScan Human | High (predicted) | PPAP2B | PLPP3 | 2.08 | Triacylglycerol Biosynthesis |
| hsa-miR-25 | miR-92a-3p (and other miRNAs w/seed AUUGCAC) | 0.03680 | -1.58 | TargetScan Human | High (predicted) | ADAMTS9 | ADAMTS9 | 2.78 | Axonal Guidance Signaling |
| hsa-miR-25 | miR-92a-3p (and other miRNAs w/seed AUUGCAC) | 0.03680 | -1.58 | TargetScan Human | High (predicted) | IFIT2 | IFIT2 | 2.97 | Activation of IRF by Cytosolic Pattern Recognition Receptors |
| hsa-miR-30a | miR-30c-5p (and other miRNAs w/seed GUAAACA) | 0.03440 | -1.54 | TargetScan Human | High (predicted) | PTPN13 | PTPN13 | 1.50 | 3-phosphoinositide Biosynthesis,3-phosphoinositide Degradation,D-myo-inositol (1,4,5,6)-Tetrakisphosphate Biosynthesis,D-myo-inositol (3,4,5,6)-tetrakisphosphate Biosynthesis,D-myo-inositol-5-phosphate Metabolism,Ephrin Receptor Signaling,Protein Kinase A Signaling,Superpathway of Inositol Phosphate Compounds |
| hsa-miR-30a | miR-30c-5p (and other miRNAs w/seed GUAAACA) | 0.03440 | -1.54 | TarBase,TargetScan Human | Experimentally Observed,High (predicted) | SLC4A7 | SLC4A7 | 1.50 |  |
| hsa-miR-30a | miR-30c-5p (and other miRNAs w/seed GUAAACA) | 0.03440 | -1.54 | TargetScan Human | High (predicted) | ERRFI1 | ERRFI1 | 1.55 | Neuregulin Signaling |
| hsa-miR-30a | miR-30c-5p (and other miRNAs w/seed GUAAACA) | 0.03440 | -1.54 | TarBase,TargetScan Human | Experimentally Observed,High (predicted) | SLC7A11 | SLC7A11 | 1.55 |  |
| hsa-miR-30a | miR-30c-5p (and other miRNAs w/seed GUAAACA) | 0.03440 | -1.54 | TargetScan Human | High (predicted) | FAM46A | FAM46A | 1.58 |  |
| hsa-miR-30a | miR-30c-5p (and other miRNAs w/seed GUAAACA) | 0.03440 | -1.54 | TarBase,TargetScan Human | Experimentally Observed,Moderate (predicted) | AADACL1 | NCEH1 | 1.68 |  |
| hsa-miR-30a | miR-30c-5p (and other miRNAs w/seed GUAAACA) | 0.03440 | -1.54 | Ingenuity Expert Findings,TargetScan Human | Experimentally Observed,High (predicted) | BCL6 | BCL6 | 1.71 | B Cell Receptor Signaling,T Helper Cell Differentiation |
| hsa-miR-30a | miR-30c-5p (and other miRNAs w/seed GUAAACA) | 0.03440 | -1.54 | TargetScan Human | High (predicted) | SOCS1 | SOCS1 | 1.78 | Acute Phase Response Signaling,Erythropoietin Signaling,Growth Hormone Signaling,IGF-1 Signaling,IL-2 Signaling,IL-4 Signaling,IL-6 Signaling,Interferon Signaling,JAK/Stat Signaling,Prolactin Signaling,Role of JAK family kinases in IL-6-type Cytokine Signaling,Role of JAK1 and JAK3 in γc Cytokine Signaling,Role of JAK1, JAK2 and TYK2 in Interferon Signaling,Role of JAK2 in Hormone-like Cytokine Signaling,Role of Macrophages, Fibroblasts and Endothelial Cells in Rheumatoid Arthritis,STAT3 Pathway,Type I Diabetes Mellitus Signaling,Type II Diabetes Mellitus Signaling |
| hsa-miR-30a | miR-30c-5p (and other miRNAs w/seed GUAAACA) | 0.03440 | -1.54 | TargetScan Human | High (predicted) | SOCS3 | SOCS3 | 1.84 | 3-phosphoinositide Biosynthesis,3-phosphoinositide Degradation,Acute Phase Response Signaling,D-myo-inositol (1,4,5,6)-Tetrakisphosphate Biosynthesis,D-myo-inositol (3,4,5,6)-tetrakisphosphate Biosynthesis,D-myo-inositol-5-phosphate Metabolism,Erythropoietin Signaling,Growth Hormone Signaling,IGF-1 Signaling,IL-10 Signaling,IL-22 Signaling,IL-6 Signaling,IL-9 Signaling,Insulin Receptor Signaling,JAK/Stat Signaling,Leptin Signaling in Obesity,Prolactin Signaling,Role of JAK family kinases in IL-6-type Cytokine Signaling,Role of JAK1 and JAK3 in γc Cytokine Signaling,Role of JAK2 in Hormone-like Cytokine Signaling,Role of Macrophages, Fibroblasts and Endothelial Cells in Rheumatoid Arthritis,STAT3 Pathway,Superpathway of Inositol Phosphate Compounds,Type I Diabetes Mellitus Signaling,Type II Diabetes Mellitus Signaling |
| hsa-miR-30a | miR-30c-5p (and other miRNAs w/seed GUAAACA) | 0.03440 | -1.54 | TargetScan Human | High (predicted) | RGS2 | RGS2 | 2.32 | cAMP-mediated signaling,G-Protein Coupled Receptor Signaling,Gαq Signaling,Gαs Signaling |
| hsa-miR-30a | miR-30c-5p (and other miRNAs w/seed GUAAACA) | 0.03440 | -1.54 | TargetScan Human | High (predicted) | ADAMTS9 | ADAMTS9 | 2.78 | Axonal Guidance Signaling |
| hsa-miR-30a | miR-30c-5p (and other miRNAs w/seed GUAAACA) | 0.03440 | -1.54 | TargetScan Human | High (predicted) | STC1 | STC1 | 2.78 |  |
| hsa-miR-30a | miR-30c-5p (and other miRNAs w/seed GUAAACA) | 0.03440 | -1.54 | TargetScan Human | High (predicted) | RASD1 | RASD1 | 3.31 | nNOS Signaling in Neurons |
| hsa-miR-424 | miR-16-5p (and other miRNAs w/seed AGCAGCA) | 0.02390 | -1.53 | TargetScan Human | High (predicted) | KCNAB1 | KCNAB1 | 1.50 |  |
| hsa-miR-424 | miR-16-5p (and other miRNAs w/seed AGCAGCA) | 0.02390 | -1.53 | TargetScan Human | High (predicted) | SLC4A7 | SLC4A7 | 1.50 |  |
| hsa-miR-424 | miR-16-5p (and other miRNAs w/seed AGCAGCA) | 0.02390 | -1.53 | TargetScan Human | High (predicted) | HSPA4L | HSPA4L | 1.51 | Aldosterone Signaling in Epithelial Cells,Protein Ubiquitination Pathway |
| hsa-miR-424 | miR-16-5p (and other miRNAs w/seed AGCAGCA) | 0.02390 | -1.53 | TargetScan Human | High (predicted) | SPRED1 | SPRED1 | 1.53 |  |
| hsa-miR-424 | miR-16-5p (and other miRNAs w/seed AGCAGCA) | 0.02390 | -1.53 | TargetScan Human | High (predicted) | RNF217 | RNF217 | 1.60 |  |
| hsa-miR-424 | miR-16-5p (and other miRNAs w/seed AGCAGCA) | 0.02390 | -1.53 | TargetScan Human | High (predicted) | SNCG | SNCG | 1.64 |  |
| hsa-miR-424 | miR-16-5p (and other miRNAs w/seed AGCAGCA) | 0.02390 | -1.53 | TarBase | Experimentally Observed | HMOX1 | HMOX1 | 2.02 | Acute Phase Response Signaling,Antioxidant Action of Vitamin C,Choline Biosynthesis III,Endothelin-1 Signaling,Fcγ Receptor-mediated Phagocytosis in Macrophages and Monocytes,Gαq Signaling,Heme Degradation,IL-10 Signaling,IL-8 Signaling,mTOR Signaling,NRF2-mediated Oxidative Stress Response,Pancreatic Adenocarcinoma Signaling,Phospholipase C Signaling,Phospholipases,Xenobiotic Metabolism Signaling |
| hsa-miR-424 | miR-16-5p (and other miRNAs w/seed AGCAGCA) | 0.02390 | -1.53 | TargetScan Human | High (predicted) | PPAP2B | PLPP3 | 2.08 | Triacylglycerol Biosynthesis |
| hsa-miR-424 | miR-16-5p (and other miRNAs w/seed AGCAGCA) | 0.02390 | -1.53 | TargetScan Human | High (predicted) | ELMOD1 | ELMOD1 | 2.23 |  |
| hsa-miR-424 | miR-16-5p (and other miRNAs w/seed AGCAGCA) | 0.02390 | -1.53 | TargetScan Human | High (predicted) | ADAMTS1 | ADAMTS1 | 2.55 | Axonal Guidance Signaling |
| hsa-miR-424 | miR-16-5p (and other miRNAs w/seed AGCAGCA) | 0.02390 | -1.53 | TarBase | Experimentally Observed | PTGS2 | PTGS2 | 2.56 | CD40 Signaling,Cholecystokinin/Gastrin-mediated Signaling,Colorectal Cancer Metastasis Signaling,Corticotropin Releasing Hormone Signaling,Eicosanoid Signaling,Endothelin-1 Signaling,Fatty Acid α-oxidation,Glucocorticoid Receptor Signaling,HGF Signaling,IL-17 Signaling,IL-8 Signaling,ILK Signaling,LXR/RXR Activation,MIF Regulation of Innate Immunity,MIF-mediated Glucocorticoid Regulation,Ovarian Cancer Signaling,Pancreatic Adenocarcinoma Signaling,PI3K/AKT Signaling,PPAR Signaling,Prostanoid Biosynthesis,Protein Kinase A Signaling,Role of IL-17A in Arthritis,Role of MAPK Signaling in the Pathogenesis of Influenza,Small Cell Lung Cancer Signaling |
| hsa-miR-424 | miR-16-5p (and other miRNAs w/seed AGCAGCA) | 0.02390 | -1.53 | TarBase, TargetScan Human, miRecords | Experimentally Observed, High (predicted) | HSPA1A | HSPA1A/HSPA1B | 3.86 | Aldosterone Signaling in Epithelial Cells,eNOS Signaling,Glucocorticoid Receptor Signaling,Huntington's Disease Signaling,Protein Ubiquitination Pathway,Unfolded protein response |
| **hsa-miR-424** | **miR-16-5p (and other miRNAs w/seed AGCAGCA)** | **0.02390** | **-1.53** | **TargetScan Human** | **High (predicted)** | **CXCL10** | **CXCL10** | **9.02** | **Agranulocyte Adhesion and Diapedesis,Communication between Innate and Adaptive Immune Cells,Granulocyte Adhesion and Diapedesis,IL-17 Signaling,IL-17A Signaling in Gastric Cells,Pathogenesis of Multiple Sclerosis,Role of Hypercytokinemia/hyperchemokinemia in the Pathogenesis of Influenza,Role of IL-17F in Allergic Inflammatory Airway Diseases,Role of MAPK Signaling in the Pathogenesis of Influenza,Thyroid Cancer Signaling,VDR/RXR Activation** |
| hsa-miR-320c | miR-320b (and other miRNAs w/seed AAAGCUG) | 0.00871 | 1.53 | TargetScan Human | High (predicted) | C9orf58 | AIF1L | -2.13 |  |
| hsa-miR-320c | miR-320b (and other miRNAs w/seed AAAGCUG) | 0.00871 | 1.53 | TargetScan Human | High (predicted) | NEDD4L | NEDD4L | -1.79 | Protein Ubiquitination Pathway |
| hsa-miR-320c | miR-320b (and other miRNAs w/seed AAAGCUG) | 0.00871 | 1.53 | TargetScan Human | High (predicted) | IRF6 | IRF6 | -1.72 |  |
| hsa-miR-1268 | miR-1268a (and other miRNAs w/seed GGGCGUG) | 0.00491 | 2.66 | TargetScan Human | High (predicted) | GGA2 | GGA2 | -1.52 |  |
| hsa-miR-1225-5p | miR-1225-5p (miRNAs w/seed UGGGUAC) | 0.00434 | 2.69 | TargetScan Human | High (predicted) | SH3BP4 | SH3BP4 | -2.04 | Clathrin-mediated Endocytosis Signaling |
| hsa-miR-1246 | miR-1246 (miRNAs w/seed AUGGAUU) | 0.00151 | 3.20 | TargetScan Human | High (predicted) | ESM1 | ESM1 | -2.17 |  |
| hsa-miR-1207-5p | miR-1207-5p (and other miRNAs w/seed GGCAGGG) | 0.00135 | 3.45 | TargetScan Human | High (predicted) | SEMA3G | SEMA3G | -1.85 | Axonal Guidance Signaling |
| hsa-miR-125a-3p | miR-125a-3p (miRNAs w/seed CAGGUGA) | 0.00006 | 4.04 | TargetScan Human | High (predicted) | AQP1 | AQP1 | -1.52 | eNOS Signaling |
